# *In silico* investigation and potential therapeutic approaches of natural products for COVID-19: Computer-aided drug design perspective

**DOI:** 10.3389/fcimb.2022.929430

**Published:** 2022-08-22

**Authors:** Md. Mominur Rahman, Md. Rezaul Islam, Shopnil Akash, Sadia Afsana Mim, Md. Saidur Rahaman, Talha Bin Emran, Esra Küpeli Akkol, Rohit Sharma, Fahad A. Alhumaydhi, Sherouk Hussein Sweilam, Md. Emon Hossain, Tanmay Kumar Ray, Sharifa Sultana, Muniruddin Ahmed, Eduardo Sobarzo-Sánchez, Polrat Wilairatana

**Affiliations:** ^1^ Department of Pharmacy, Faculty of Allied Health Sciences, Daffodil International University, Dhaka, Bangladesh; ^2^ Department of Pharmacy, BGC Trust University Bangladesh, Chittagong, Bangladesh; ^3^ Department of Pharmacognosy, Faculty of Pharmacy, Gazi University, Ankara, Turkey; ^4^ Department of Rasashastra and Bhaishajya Kalpana, Faculty of Ayurveda, Institute of Medical Sciences, Banaras Hindu University, Varanasi, Uttar Pradesh, India; ^5^ Department of Medical Laboratories, College of Applied Medical Sciences, Qassim University, Buraydah, Saudi Arabia; ^6^ Department of Pharmacognosy, College of Pharmacy, Prince Sattam Bin Abdulaziz University, Al-Kharj, Saudi Arabia; ^7^ Department of Pharmacognosy, Faculty of Pharmacy, Egyptian Russian University, Badr City, Egypt; ^8^ Instituto de Investigación y Postgrado, Facultad de Ciencias de la Salud, Universidad Central de Chile, Santiago, Chile; ^9^ Department of Organic Chemistry, Faculty of Pharmacy, University of Santiago de Compostela, Santiago de Compostela, Spain; ^10^ Department of Clinical Tropical Medicine, Faculty of Tropical Medicine, Mahidol University, Bangkok, Thailand

**Keywords:** virtual screening, drug design, COVID-19, natural products, alkaloids, SARS-CoV-2

## Abstract

The severe acute respiratory syndrome coronavirus 2 (SARS-CoV-2) has caused a substantial number of deaths around the world, making it a serious and pressing public health hazard. Phytochemicals could thus provide a rich source of potent and safer anti-SARS-CoV-2 drugs. The absence of approved treatments or vaccinations continues to be an issue, forcing the creation of new medicines. Computer-aided drug design has helped to speed up the drug research and development process by decreasing costs and time. Natural compounds like terpenoids, alkaloids, polyphenols, and flavonoid derivatives have a perfect impact against viral replication and facilitate future studies in novel drug discovery. This would be more effective if collaboration took place between governments, researchers, clinicians, and traditional medicine practitioners’ safe and effective therapeutic research. Through a computational approach, this study aims to contribute to the development of effective treatment methods by examining the mechanisms relating to the binding and subsequent inhibition of SARS-CoV-2 ribonucleic acid (RNA)-dependent RNA polymerase (RdRp). The *in silico* method has also been employed to determine the most effective drug among the mentioned compound and their aquatic, nonaquatic, and pharmacokinetics’ data have been analyzed. The highest binding energy has been reported -11.4 kcal/mol against SARS-CoV-2 main protease (7MBG) in L05. Besides, all the ligands are non-carcinogenic, excluding L04, and have good water solubility and no AMES toxicity. The discovery of preclinical drug candidate molecules and the structural elucidation of pharmacological therapeutic targets have expedited both structure-based and ligand-based drug design. This review article will assist physicians and researchers in realizing the enormous potential of computer-aided drug design in the design and discovery of therapeutic molecules, and hence in the treatment of deadly diseases.

## 1 Introduction

The novel coronavirus pandemic originated in December 2019 in Wuhan, Hubei Province, China (Omrani et al., 2020), which is the biggest current threat worldwide, and the number of cases is still growing (Shang et al., 2020), spread by cells within the bodies of those infected (Sigrist et al., 2020). It is recognized as a pandemic that has severe morbidity and mortality levels by the World Health Organization (WHO) on March 11, 2020 (Luo et al., 2020a). The COVID-19 disease mainly occurrs through a coronavirus that belongs to the single-strand RNA (Hadi et al., 2020) β-coronavirus family (Control and Prevention, 2020). SARS-CoV-2 primarily exists in a state for up to 72 hours in which it can be transmitted easily (Ardalan et al., 2014). Respiratory droplets can facilitate a significant transmission route to the person in any indoor environment initiated by sneezing, coughing, or speaking. It is one of the most contagious diseases in that it can spread before identifying signs and symptoms. Complications from COVID-19 may include shortness of breath, pneumonia, fever, dry cough, diarrhea, fatigue, sore throat, nose congestion, and acute respiratory distress syndrome that leads to septic shock and finally death (Bridwell et al., 2020; Monteleone et al., 2020).

Recent research suggests that lung inflammation and airway damage contribute to morbidity and mortality due to SARS –CoV-2 (Amaral-Machado et al., 2020). The mortality in COVID-19 occurs due to the extreme release of cytokines, leading to the inflammation of lung tissue (Long et al., 2020; Terpos et al., 2020). Covid infection initiates from interaction with Angiotensin-converting enzyme (ACE2) receptors and transmembrane protease serine 2 (TMPRSS) on the host cell membrane. However, preventive approaches, including maintaining distance, washing hands, and wearing masks, can reduce the morbidity and mortality rate of COVID-19 (Gomez et al., 2021). Several vaccine candidates are being developed, but it is challenging as they contain nucleotide and protein-based components that require sensitive manufacturing and storage conditions. Faced with this enormous worldwide burden, we are working to develop a COVID-19 treatment that can be mass-produced and supplied swiftly. Natural compounds, which often have low toxicity and are used in the pharmaceutical sector for their bioactivity, including antiviral activity, could give a solution to this conundrum. Because SARS-CoV-1 and COVID-19 are so similar, it could lead to the development of new treatments or perhaps a vaccine. The anti-SARS-CoV-2 activity of flavanols, flavanones, and flavanols, as well as the fact that these metabolites are abundant in angiosperm plants, have given rise to a lot of hope. Because the majority of current research is theoretical or lacks analytical confirmation, there is still a long way to go in terms of biological analysis and efficient extraction and manufacturing (da Silva Antonio et al., 2020b). Medicine discovery is a lengthy process that can take up to 10-15 years (Hassan Baig et al., 2016) and can cost up to 2.558 billion dollars to bring a drug to market (Dimasi et al., 2016). It is a multistep process that begins with the identification of a viable therapeutic target, followed by drug target validation, hit to lead discovery, lead molecule optimization, and preclinical and clinical research (Gurung et al., 2021).

Despite the significant financial and time commitments required for medication development, clinical trial success is just 13 percent, with a high drug attrition rate (Zhong et al., 2018). Drug failure has been observed in the majority of instances (40-60%) due to a lack of optimal pharmacokinetic features on absorption, distribution, metabolism, excretion, and toxicity (ADME/Tox) (Hou and Xu, 2004). Leading pharmaceutical corporations and research organizations have used computer-aided drug discovery (CADD) tools in preliminary investigations to help speed up the drug discovery and development process, reducing costs and failures in the final stage (Yu and MacKerell, 2017). The use of rational drug design as part of CADD can help researchers better understand the binding affinity and molecular interaction between the target protein and the ligand. Furthermore, the availability of supercomputing facilities, parallel processing, and improved programs, algorithms, and tools has aided leading discoveries in pharmaceutical research (Macalino et al., 2015). The genome of SARS-CoV-2, like that of SARS and Middle East respiratory syndrome coronavirus (MERS), encodes sixteen nonstructural proteins (nsps) such as main protease (Mpro), papain-like protease, RNA-dependent RNA polymerase (RdRp), helicase, and other accessory proteins, as well as four structural proteins (envelope, membrane, spike, and nucleocapsid). While the spike glycoprotein is required for virus contact with the host cell receptor, the nsps play an important role in the virus life cycle by producing subgenomic RNAs (Dai et al., 2020; Goyal and Goyal, 2020). As a result, nonstructural and structural proteins are interesting targets for the design and development of COVID-19 antiviral medicines (Goyal and Goyal, 2020). The lack of effective COVID-19 vaccines or medications, as well as the high death rate, necessitates the quick finding of innovative therapies (Tu et al., 2020), and computer-aided drug design is thought to be a significant technique for identifying novel therapeutics. Using natural lead compounds derived from virtual screening and pharmacokinetic prediction (Gopal and Skariyachan, 2020), it is possible to produce effective lead molecules against COVID-19. Due to the availability of pharmacokinetic and pharmacodynamic data for broad-spectrum antiviral medicines, repurposing is a promising technique for speeding up the development of a potential treatment for SARS-CoV-2 infection in humans (Pillaiyar et al., 2020).

As natural products and herbal medicines have a promising contribution to drug discovery, these are used to prevent COVID-19 infection (Bogan-Brown et al., 2021). Plants, fungus, and marine sources of natural products are already used to develop drug discovery for several diseases. These show antiviral effects against human CoV-2 that will aid the development of a noble drug for the treatment of COVID-19 (Huang et al., 2020b). These natural products worked by inhibiting the interaction between the S protein and the ACE2 receptors of host cells. Many of these boost the immune system to protect against the respective virus, fibrosis, oxidative stress, and inflammation associated with COVID-19. Natural products can be used in combination with the clinical standard care for practical outputs. In elderly patients and patients at an advanced stage, natural products can suppress the disease progression and reduce complications and mortality (Huang et al., 2020a).

In this review, we aimed to deliver new aspects with natural products for the treatment and prevention of COVID-19 (Huang et al., 2020). Natural products also contribute to developing novel drug discovery, so taking a preventive approach by natural products must be practical and have potential (Janeway Jr, 2001; Ho et al., 2007). Ultimately this integrated analysis will present promising evidence regarding the treatment and prevention of COVID-19 by natural products (Nassiri‐Asl and Hosseinzadeh, 2009; Choi et al., 2017; Laurent et al., 2017; Omrani et al., 2020). The complete genome sequence of the severe acute respiratory syndrome coronavirus 2 (SARS-CoV-2) and the elucidation of viral protein structures using X-ray crystallography, nuclear magnetic resonance (NMR), electron microscopy, and a homology modeling approach have enabled the identification of inhibitor drugs against COVID-19’s essential therapeutic drug targets.

## 2 Epidemiology

Infectious disease transmission is driven by three key factors: transmission pathways, infection sources, and vulnerable hosts. According to the National Health Commission of China’s most recent reports, SARS-CoV-2 is passed on to people via respiratory droplets, close contact, and surface contamination, with aerosol transmission potentially being an option (Wu et al., 2020a).

Previous epidemics of SARS (2003) and MERS (2012) have shown that family members can trigger significant outbreaks in the hospital. Research with SARS has found that managing patients both inside and outside the hospital is one of the critical phases in disease control. For example, in Vancouver (Canada), many import cases were managed and diagnosed quickly, and secondary transmission was avoided in town. The inefficient control of diseases in Toronto, Canada, and Taipei, Taiwan has led to the transmission and hospitalization of serious diseases [25]. SARS-CoV-2 was labeled the sixth international public health concern and pandemic by the World Health Organization (WHO) on 11 March 2020 (Colson et al., 2020; Wu et al., 2020a). As of November 2020, 46 million confirmed cases and one million deaths had been registered worldwide, according to current laboratory tests. According to a WHO report issued on November 2, the most prevalent cases were identified in America (20,807,415), Asia (13,626,009), Europe (10,324,515), Africa (1,796,748), and Oceania (which included Australia, French Polynesia, Guam, New Zealand, and Papua New Guinea) (41,916). (World Health Organization, Coronavirus Disease, 2020). Several studies have revealed SARS-CoV-2 death rates. It is expected to be 6.9 percent of confirmed cases. However, this can vary considerably (Wu et al., 2020a). Singapore, for example, has a rate of 0.1 percent, while Belgium has a rate of 15.4 percent (Johns Hopkins University). SARS-CoV-2 has a lower mortality rate than SARS-CoV or MERS-CoV, with rates of 10% and 37.1 percent, respectively, but it is ten times more contagious than the other viral illnesses (Lescure et al., 2020). According to statistical methods, the infectious rate of SARS-CoV-2, denoted as R0, is approximately between 1.3 and 6.5, with an average of 3.3 (Sen-Crowe et al., 2020). Recent reviews involve 212 studies from 11 countries with 281,461 individuals at an average age of about 45 having been analyzed. Studies showed a severe disease rate of 23% and a mortality rate of 6%. Among these, the highest severe disease rate is 38% in Wuhan, China, and the highest mortality rate is 14% in Italy. Moreover, hypertension, diabetes, malignancy are more likely to exist in severe COVID-19 patients (Li et al., 2021).

Many conclusions suggest that SARS-CoV-2 has a long time of incubation (2 to 14 days) and a significant potential for asymptomatic communication that might lead to the fast transmission of the virus (Ahn et al., 2020). The outbreak’s pattern suggests that people of all ethnicities and ages are susceptible to the sickness. The elderly and persons with underlying conditions such as cardiovascular disease, diabetes, and high blood pressure, on the other hand, have a higher rate of severe illness and fatality (Wu et al., 2020c). COVID-19 Epidemiology highlights the infection load, transmission dynamics, and other epidemiological aspects. While infection rates are notably high in China, Italy, and the United States, the disease is progressively spreading in India as well, endangering the country’s health and economy. The rapid spread of infection has been attributed to asymptomatic transmission, the early symptomatic phase, and restricted access to testing in various contexts. According to a large case series from China, 81% of the cases had mild symptoms, 14 percent had severe disease, and 5% had critical illness. While China’s death rate was estimated to be 2.3 percent, Italy, which has a large senior population, reported a case fatality rate of 7.2 percent due to greater infection and mortality rates among the elderly. COVID-19 is a highly contagious disease that affects a considerable number of healthcare workers, as indicated by the fact that healthcare workers accounted for a significant share of reported infections in the United States. Providing health care for COVID-19 patients as well as those with other acute and chronic conditions who have limited access to healthcare facilities and services is a challenge for low- and middle-income countries’ health systems, which necessitates immediate health system strengthening across sectors (Aovi et al. 2021; Rafiq, 2021).

## 3 Potential therapeutic approach against COVID-19

Several vaccines and pharmacologic treatment approaches are being developed, but natural sources like plants and fungi may have some potential effect against COVID-19 infection (Lin et al., 2014). Viral internalization in host cells is strongly related to the cellular ACE2 enzyme surface spike protein virus. The mechanism of viral entry to host cells is connected to two routes like endocytic uptake and viral membrane in fusion (Wang et al., 2008). Viral S protein, activated by host cell protease serine 2 (TMPRSS2), facilitated viral infection into host cells and allowed the release and replication of viral genomic RNA. Fusing to host cells, receptor-mediated endocytosis permits the uptake of viral particles into cells followed by the cleavage of the S-protein due to cathepsin L inside the endosome to express viral RNA genome into host cells.

**Figure 1:**
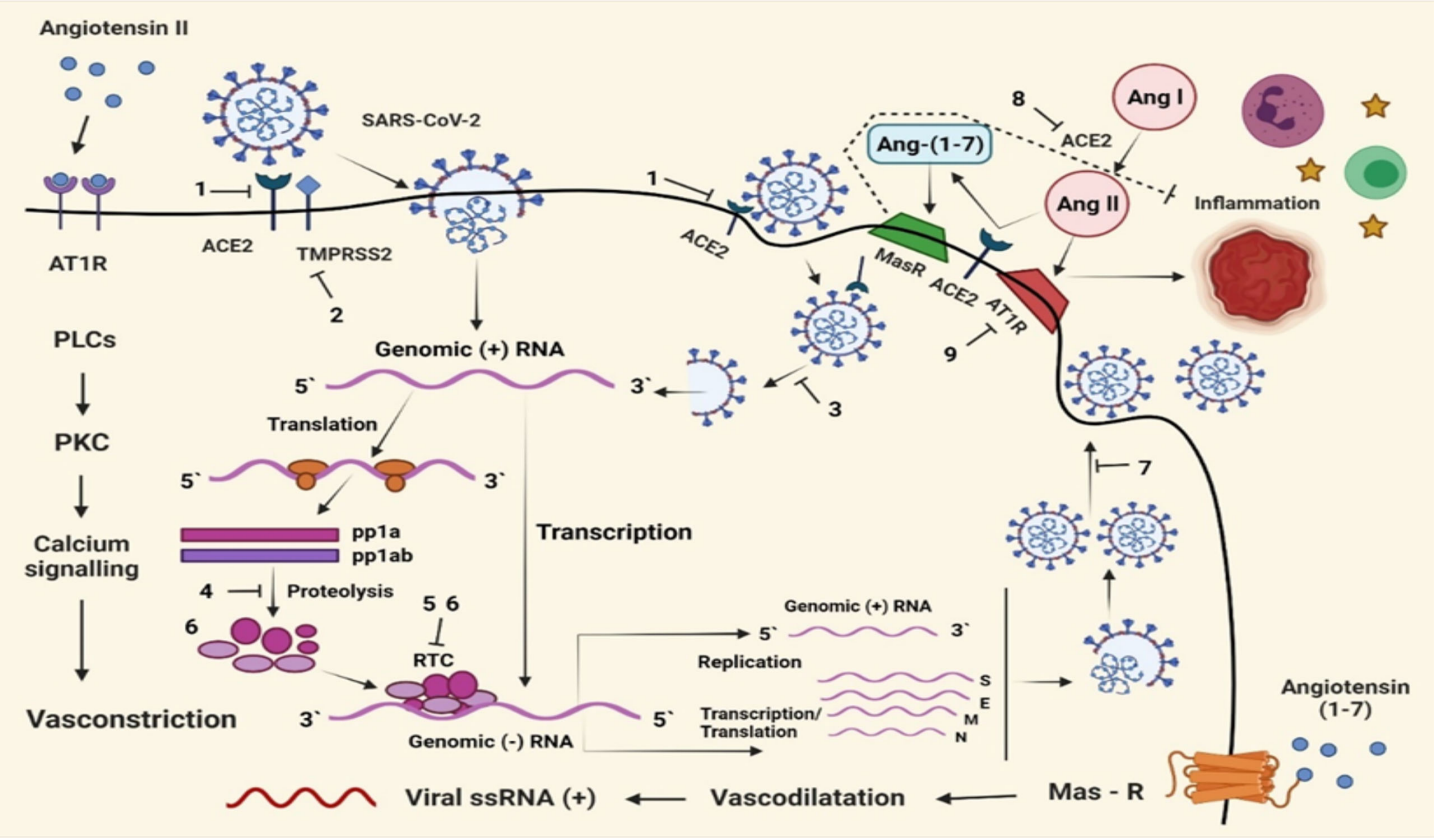
Potential therapeutic strategies are concerned with four aspects: inhibition of virus entry, virus replication, the release of virus offspring, and modifying RAAS. Viral S-protein bonding to ACE2 1. Activity of TMPRSS; 2. Endocytic pathway-Cleavage of S-protein by cathepsin L; 3. Internalization of virus prevented; 4. The replicative activity of RTC inhibited; 5. Increase intracellular Zn^2+^ concentration; 6.Inhibition of viroporin mediated viral budding; 7. Blockage of ACE2 resulting in excessive inflammation; 8. AT1R; 9. 3CL^Pro^,3 -chymotrypsin like protease; ACE2; AT1R; S, spike; N, nucleocapsid; E, envelop; M, membrane; PL^Pro^, pepsin like protease; PP, polyprotein; RAAS; RTC, replication-transcription complex; TMPRSS2, transmembrane protease serine 2 (Prasansuklab et al., 2020).

Furthermore, offspring of the virus inside host cells may be initiated by translation of viral genome into two precursor polyproteins pp1a and pp1ab that will further cleavage to nonstructural proteins by encoded protease enzyme that ultimately is used for application of transcription complex for producing genomic RNA and subgenomic mRNA. The m-RNA translated into structural proteins with viral genome initiated new virions and released through viroporin-mediated viral budding (Lu et al., 2006). Blood pressure inflammation fibrosis regulated by RAAS (Renin Angiotensin Aldosterone System) where ACE2 works by converting angiotensin I to angiotensin II, which is further converted to lung-protective angiotensin (Mehta et al.; Xu et al.; Voeltz et al., 2006; Bai et al., 2020; Morawska and Cao, 2020; South et al., 2020; Van Doremalen et al., 2020; Walls et al., 2020). SARS-CoV-2 binds to ACE2 to enter into cells, so that ACE2 cannot work properly. Angiotensin II binds to Angiotensin II type 1 receptor (AT1R) instead of converting angiotensin (1-7), which causes inflammation and disturbance in the renal and cardiovascular system (**Figure 1**) (Shete, 2020). So therapeutic approaches should be concerned about the mechanism innovation of viral programming and regulating the immune system with RAAS (Prasansuklab et al., 2020).

## 4 Prevention of COVID-19 and immune enhancer

The immune system plays a vital role against infectious pathogens to protect our body (Voeltz et al., 2006). An older person is more likely to have an suppressed immune system, so they are at a higher risk of being affected by COVID-19 (McIntosh et al., 2020). Ensuring the intake of a sufficient amount of nutrients is the first choice of methods to boost immune system function and prevent COVD-19 disease. Scientific evidence shows that proper diet modification and a healthy lifestyle can aid in a preventive approach for the COVID-19 pandemic (Zildzic et al., 2020). Various fresh animals contain micronutrients like vitamin A, D, E, C, B6, B12 and trace elements like zinc, selenium, and iron that can improve immune function and fight against infectious diseases (Chaplin, 2010; Elmadfa and Meyer, 2019). As COVID-19 mainly weakens the immune response, the survival rate depends on the strength of immunity. Balanced nutritional supplements provide a strong immunity, ensuring the body’s defense mechanism against infection (Miles et al., 2020). Research evidence shows that micronutrients and iron, zinc, and selenium provide a synergistic effect on the body’s immunity function (Diwakar et al., 2016) (Weiss, 2002; McClung and Peterson, 2010; Haryanto et al., 2015; Saeed et al., 2016) and inhibit viral replication inside host cells (Chaturvedi et al., 2004). Folate, copper, and selenium can increase antibody production (Haryanto et al., 2015) and cell and antibody-mediated immune response (Saeed et al., 2016) by activating cytokines and chemokines (Maggini et al., 2007). Several micronutrients, especially omega-3 fatty acids, eicosatetraenoic acid, and docosahexaenoic acid, develop the immune system’s ability to fight against viral infection (Calder et al., 2020).

A variety of nutraceuticals have been shown to have immune-boosting, antiviral, antioxidant, and anti-inflammatory properties. Zn, vitamin D, vitamin C, curcumin, cinnamaldehyde, probiotics, selenium, lactoferrin, quercetin, and other nutrients are among them. Combining some of these phytonutrients in the right combination as a food supplement may help to boost the immune system, prevent virus spread, prevent disease progression to a severe stage, and suppress hyper inflammation even further, providing both prophylactic and therapeutic support against COVID-19 (Mrityunjaya et al., 2020; Ahmed et al., 2021). Micronutrients stimulate the immune system and can be used as a treatment for those who do not want to use drugs. Micronutrient insufficiency leads to immune system dysfunction. It suggests that eating foods high in vitamins C, D, and E, as well as minerals like zinc, can help boost immunity and help fight diseases caused by bacteria, viruses, and parasites. Flavonoids have been identified as potential COVID-19 transmission inhibitors in several investigations. Vitamins C, D, and E, as well as zinc and flavonoids, have been proven to have strong correlations to boosting the immune system. Individuals and populations may be protected from acquiring a severe disease as a result of this, which has an impact on the COVID-19 disease process. With these established links, it would be useful to implement dietary advice during the pandemic to lower the incidence of these chemical deficiencies. This will lower the risk of serious disease and, more importantly, increase the chances of survival.

## 5 Natural products for inhibiting COVID-19

### 5.1 Natural products inhibiting MERS-CoV

Several tests examined the possibility of ordinary things being rectified by MERS-CoV. A more efficient MERS-CoV reproduction inhibitor was Silvestro, an Aglaia sp. Phytochemicals (EC50 of 1.3 nM). Silvestro is an RNA helicase eIF4A inhibitor that hinders viral replication while diminishing CoV protein creation and obstructing replication/record buildings (Müller et al., 2018). Quite possibly, the most encouraging inhibitor of MERS-CoV is Griffithsin, a 12.7 kDa lectin found in the Griffithsia species (red-green growth). It has three sugar restricting areas that permit it to tie accurately to glycans on CoV protein spikes and forestall viral connection to have cells, with extraordinary strength exhibited in vitro concentrates against MERS-CoV (EC50 of 0.125 M) and various CoV strains (EC50 of 0.00320.33 M) (Millet et al., 2016). Griffithsin additionally seems to have a low foundational poisonousness, with an explicitness list of 30–3100 against HcoV cells (contrasted with human colorectal adenocarcinoma or fibroblast cell lines) (O’Keefe et al., 2010), demonstrating that it very well may be one of the leading contenders for the animal and clinical preliminaries against SARS-CoV-2 (Mani et al., 2020).

### 5.2 Natural products inhibiting SARS-CoV

#### 5.2.1 Polyphenols

Polyphenols have been displayed to have antiviral movement in various examinations. Quercetin, for instance, has an IC50 of 8.6 3.2 M against SARS-CoV papain like protease (PLpro) (Park et al., 2017). There was no antiviral action test done on cells. Quercetin is a flavonoid found in various food varieties. However, it is particularly bountiful in specific berries and spices (Kaack and Austed, 1998; Justesen and Knuthsen, 2001). As recently referenced, the related polyphenolics myricetin and scutellarein have moderate inhibitory impacts against SARS-CoV helicase [64]. Poly phenolics were distinguished as the bioactive segments responsible for the adequacy of this plant species against SARS-CoV. Bavachinin, neobavaisoflavone, isobavachalcone, 4′-O-methylbavachalcone, psoralidin, and corylifol A were likewise recuperated from the ethanolic separates, with antiviral movement going from 4.2 to 38.4 M. There were no cell-based antiviral tests done this time. Isobavachalcone and psoralidin had the best antiviral action, and both were found to be blended, reversible inhibitors of PLpro by means of a sort I component (restricting to the free catalyst instead of the protein substrate complex) (Kim et al., 2014).

#### 5.2.2 Lectins

Plant lectins, which are proteins that tightly bind to starch bunches specifically and reversibly (Mitchell et al., 2017), are another regularly ccurring synthetic option that may stifle SARS-CoV. Lectins’ antiviral properties have been shown against infections, such as flu and herpes simplex disease (Hwang et al., 2020), as well as Ebola (Michelow et al., 2011; Covés-Datson et al., 2019). Shockingly, animals that had their plasma levels of recombinant human mannose-restricting lectin raised had the option to endure, in any case, deadly Ebola contaminations (Michelow et al., 2011). Utilizing an examination of the cytopathic impact effect (CPE), Keyaerts et al. (2007) tried the action of a broad scope of plant lectins (33 taking all things together) against SARS-CoV, getting EC50 esteems as low as 0.45 0.08 g/mL for Lycoris radiata agglutinin. Although the specific mechanism of action still can not seem to be found, the most viable targets were believed to be enacted at the phase of viral connection or towards the finish of the irresistible viral cycle. Different lectins have shown moderate to excellent resistance in human preliminaries (Petersen et al., 2006), so they could be one of the additional promising classes of typically inferred compounds to treat SARS-CoV-2 and other Covid diseases with more exploration.

#### 5.2.3 Terpenoid derivatives

Glycyrrhiza glabra (Leguminosae) or its bioactive constituents, glycyrrhizin, have antiviral efficacy against a variety of pathogens, like viruses such as Hepatitis A, Hepatitis B, and Hepatitis C, dangerous varicella-zoster virus, Human immunodeficiency virus (HIV), HS (herpes simplex) type-1, and Cytomegalovirus (Asl and Hosseinzadeh, 2007). A medical experiment in 2003 found that glycyrrhizin has antimicrobial properties, specifically antiviral, against two clinical isolates of coronavirus (FFM-1 and FFM-2) from sufferers. Glycyrrhizin suppressed the replication of SARS-related virus and could be considered for SARS therapy (Cinatl et al., 2003). Additionally, an in vitro investigation revealed that glycyrrhizin had antiviral properties against a virus named SARS (Chen et al., 2004). Quinone-methide triterpenoids are a type of terpenoid found only in the Celastraceae family, like Tripterygium regalia. With an IC50 of 2.6–10.3 M, those drugs displayed a modest suppressing effect towards 3-chymotrypsin like protease (3CLpro). As per structure activity relationship (SAR) studies, the quinone-methide molecule plays an essential part in 3Clpro suppression (Ryu et al., 2010b). S. miltiorrhiza produces tanshinones having an abietane diterpene backbone. Tanshinones have such a diversity of physiological actions, including anti-inflammatory, cardiac, and anticancer properties.

#### 5.2.4 Alkaloid derivatives

Lycoris radiata (Amaryllidaceae) extracts possess significant antimicrobial activities against SARS-CoV, according to a 2005 study employing the MTS test for virus-induced cytopathogenic impact. The main ingredient within that sample is lycorine, an alkaloid backbone with an EC50 score of 15.7±1.2 nM, indicating strong antimicrobial properties. These findings show that lycorine is a promising compound for developing novel antiviral drugs (Li et al., 2005). The additional survey suggests that lycorine can suppress the reproduction of COVID-19 virus-like HcoV-OC43 (EC50: 0.15 M), MERS-CoV (EC50: 1.63 M), and HcoV-NL63 (EC50: 0.47 M) in vitro. Lycorine can also lower infection rates in the CNS of BALB/c mice or save them from HcoV OC43-induced death (Shen et al., 2019). Carapichea ipecacuanha root (Rubiaceae) contains Emmeline, an alkaloid that has anti-protozoal and repellent properties. Emetine inhibited HcoV-OC43 (EC50: 0.30 M), MERS-CoV (EC50: 0.34 M), and HcoV-NL63 (EC50: 1.43 M) COVID-19 replication in a laboratory. Apart from this, Emmeline can also prevent MERS-CoV from infecting viral DNA (Weber and Opatz, 2019). Stephania tetrandra (Menispermaceae) roots contain bisbenzyl isoquinoline alkaloids with antitumor, anti-inflammatory, and antioxidative properties (Kim et al., 2019a). Tetrandrine (IC50: 14.51 M), fangchinoline (IC50: 12.40 M), and cepharanthine (IC50: 10.54 M) are the major ingredients of S. tetrandra alkaloids that demonstrate potent antimicrobial efficiency against HcoV-OC43 contamination and copy of viral DNA suppression (Nassiri‐Asl and Hosseinzadeh, 2009).

### 5.3 Natural products inhibiting SARS-CoV-2

Given the brief time frame since its rise, not many examinations have been distributed on SARS-CoV-2. Be that as it may, some exploration (Toney et al., 2004; Liu and Zhou, 2005; Wang et al., 2007; Lung et al., 2020; Zhang et al., 2020a) has investigated the utilization of computer modeling for screening purposes. Commonly, these models ascertain the free restricting energy between a ligand and a receptor (Forli et al., 2016), with a lower free restricting power inferring a more grounded ligand-receptor association. Even though it may be hard to obtain comparative outcomes utilizing distinctive displaying procedures (Aldeghi et al., 2016), computer modeling empowers researchers to assess the overall restricting partiality of a gathering of mixtures to the receptor. The speed and adaptability of this innovation might be beneficial for rapidly tracking down an incredible inhibitor of SARS-CoV-2, as well as bringing down the significant expenses and period associated with actually screening enormous banks of synthetic compounds or plant separates for bioactivity (Chen et al., 2017). Mixtures that utilize this strategy would then be tried in cell-based examinations to perceive how compelling and safe they are in vitro before continuing to the animal and clinical testing tried 83 substances present in customary Chinese prescriptions for activity against SARS-RNA-subordinate CoV-2’s RNA polymerase, distinguishing theaflavin, a cancer prevention agent polyphenol, as a potential inhibitor. Additionally, a report carefully assessed 115 substances revealed in Chinese traditional medicines and chose 13 for additional exploration (Zhang et al., 2020a). A few of these were polyphenolic compounds found in nature, for example quercetin and kaempferol, which have effectively started considering the treatment of different infections (**Table 1**) (Tome-Carneiro and Visioli, 2016; Khan et al., 2019; Cassidy et al., 2020).

**Table 1:** List of compounds derived from natural sources that are potential anti-COVID-19 drug candidates (Prasansuklab et al., 2020).

Plant/Family	Product	Dosage/Duration	Model/Strains	Efficacy	Reference
Alnus japonica (Thunb.) Steud. (Betulaceae)	Hirsutenone (Ethanol extract)	0-200 µM/60min	In vitro SARS-CoV-Plpro	IC50 = 4.1 ± 0.3 µM	(Park et al., 2012a)
Angelica keiskei (Miq.) Koidz (Umbelliferae)	Xanthoangelol E (Ethanol extract)	0, 12.5, 25, 50 µM	In vitro SARS-CoV-PLpro SARS-CoV-3CLpro	Activity inhibition of SARS-CoV-PLpro activity (IC50 ¼ = 1.2 ± 0.4 µM) Inhibition of SARS-CoV-3CLpro activity (IC50 = 11.4 ± 1.4 µM)	(Park et al., 2016)
Aglaia perviridis Hiern (Meliaceae)	Myricetin	0.01-10 µM	In vitro angiotensin converting enzyme from rabbit lung	IC50 = 2.71 ± 0.19 µM	(Yu et al., 2012)
Cibotium barometz (L.) J.Sm. (Dicksoniaceae)	Ethanol and methanol extract	0, 25, 50, 100, and 200 µ/mL	In vitro SARS-CoV Virus propagated in Vero E6 cells	EC50 = 8.42 and ≥ 10 µg/mL	(Wen et al., 2011)
Cullen corylifolium (L.) Medik. (Leguminosae)	Psoralidin (Ethanol extract)	0-100 µM	In vitro SARS-CoV-PLpro	IC50 = 4.2 ± 1.0 µM	(Kim et al., 2014)
Ecklonia cava (Laminariaceae)	Dieckol (Ethanol extract)	0-200 µM	In vitro SARS-CoV-3CLpro	IC50 = 2.7 ± 0.6 µM	(Park et al., 2013)
Paulownia tomentosa (Thunb.) Steud. (Scrophulariaceae)	Tomentin E (Methanol extract)	0, 6.25, 12.5, 25 µM	In vitro SARS-CoV-PLpro	IC50 = 5.0 ± 0.06 µM	(Cho et al., 2013)
Quercus infectoria G. Olivier (Fagaceae)	Ethanol-water extract	330 µ/mL	In vitro	Inhibition of ACE activity by 93.3 ± 2.5%	(Sharifi et al., 2013)
Rheum sp. Polygonum sp. (Polygonaceae)	Emodin	0, 10, 50, 100, 200 and 400 µ/mL	In vitro Vero cells	IC50 = 200 µM	(Ho et al., 2007)
Salvia miltiorrhiza Bunge (Lamiaceae)	Cryptotanshinone (n-Hexane extract)	0-200 µM/30min	In vitro SARS-CoV-Plpro	IC50 = 0.8 ± 0.2 µM	(Park et al., 2012b)
	Dihydrotanshinone l (n-Hexane extract)	0-200 µM/60min	In vitro SARS-CoV-3CLpro	IC50 = 14.4 ± 0.7 µM	
Sambucus javanica subsp. Chinensis (Lindl.) Fukuoka (Adoxaceae)	95% ethanol extract	0, 1, 10, 50 µg/mL/36h	In vitro HcoV-NL63 inLLC-MK2 cells	Inhibition of viral cytopathicity – (IC50 = 1.17 ± 0.75 µg/mL) Inhibition of viral attachment (IC50 = 15.75 ± 6.65 µg/mL)	(Weng et al., 2019)
	Caffeic acid	0, 10, 50,100 µM/36h	–	Inhibition of viral cytopathicity – (IC50 = 3.54 ± 0.77 µM)	
	Chlorogenic acid	–	–	Inhibition of viral cytopathicity – (IC50 = 43.45 ± 6.01 µM)	
	Gallic acid	–	–	Inhibition of viral cytopathic – (IC50 = 71.48 ± 18.40 µM)	
Scutellaria baicalensis Georgi (Labiatae)	Scutellarein	0.01-10 µM	In vitro	IC50 = 0.86 ± 0.48 µM	(Yu et al., 2012)
Torreya nucifera (L.) Siebold & Zucc. (Taxaceae)	Amentoflavone (Ethanol extract)	0-300 µM	In vitro SARS-CoV-3CLpro	IC50 = 8.3 ± 0.6 µM	(Ryu et al., 2010a)
Tribulus terrestris L. (Zygophyllaceae)	Terrestrimine (Methanol extract)	1,10,100,1000 µM	In vitro SARS-CoV-PLpro	IC50 = 15.8 ± 1.2 µM	(Song et al., 2014)
	Lianhuaqingwen (Herbal mixture) dissolved in DMSO and then in serum-free DMEM	0-600 µg/mL/72h	In vitro SARS-CoV-2 Virus propagated in Vero E6 cells	IC50 = 412.2 µg/mL	(Runfeng et al., 2020)
	Herbacetin	1, 2.5, 20 µM/16h	In vitro SARS-CoV-3CLpro	IC50 = 33.17 µM	(Jo et al., 2020)
	Pectolinarin	1, 2.5, 20 µM/16h	In vitro SARS-CoV-3CLpro	IC50 = 37.78 µM	(Jo et al., 2020)
	Rhoifolin	1, 2.5, 20 µM/16h	In vitro SARS-CoV-3CLpro	IC50 = 27.45 µM	(Jo et al., 2020)

#### 5.3.1 Camel milk

Camel milk contains various antimicrobial and immunological proteins and enzymes that defend against bacterial and viral infections. Immunoglobulins, lactoferrin, lysozyme, lactoperoxidase, peptidoglycan recognition protein, vitamins C, and oligosaccharides are some examples of antimicrobials (Mehta and Agrawal, 2020). However, lactoferrin and immunoglobulins are responsible for most camel milk’s antimicrobial and antiviral properties. Lactoferrin from camel milk behaves differently and uniquely than lactoferrin from other mammals’ milk, although the inhibitory mechanism is similar to lactoferrin from bovine milk (Badr et al., 2017). Lactoferrin boosts the immune system by defending host cells from bacterial, viral, and inflammatory infections (Mohammadabadi and Hussain, 2021).

SARS-spike CoV-2’s protein allows the virus to enter host cells. Hence increasing the immune system will be beneficial in combating this virus. Various protective proteins and enzymes, such as lactoferrin, are found in cattle milk, particularly camel milk, and have immunological capabilities against bacterial and viral illnesses. Lactoferrin from milk contains immunomodulatory qualities that help the host’s immune system respond better and prevent infections. Nutritional supplements are effective against COVID-19, but there have been few clinical trials (Bruni et al., 2016). Lactoferrin is an antiviral factor that protects against viruses like SARS-CoV (Chen et al., 2020b). Lactoferrin may block SARS-CoV-2 invasion the same way that SARS-CoV does [109] because 79 percent of the sequences of SARS-CoV and SARS-CoV-2, as well as the receptor-binding domain, are similar. Without breathing assistance, the frequency of COVID-19 in babies was low, and lower respiratory tract infections were uncommon (Peroni and Fanos, 2020). Lactoferrin blocked virus entrance in human coronaviruses hCOV-NL63 and pseudotyped SARS-CoV by attaching to heparan sulfate glycosaminoglycan on the cell surface (Lang et al., 2011). Although there are no published studies on the effects of lactoferrin on SARS-CoV-2 entry into host cells, the interaction of lactoferrin with heparan cell receptors, which allows virus attachment on the cell surface in the primary phase of infection, particularly in coronaviruses (Mehta and Agrawal, 2020), has been studied.

Lactoferrin suppresses virus buildup on cell surfaces, inhibits virus-host cell interaction, and prevents viral infection, as seen in the SARS-CoV epidemic and maybe also in SARS-CoV-2. A viral infection causes mortality in some severe COVID-19 cases and elevations in cytokines and acute phase reactants such as interleukin IL-6, tumor necrosis factor-a (TNFa), and ferritin (Rosa et al., 2017). Lactoferrin inhibited the human coronavirus of pseudotyped SARS-CoV, most closely related to SARS-CoV-2, which produces COVID-19, by 50%. Lactoferrin from milk is beneficial in the innate response to diseases like SARS-CoV-2. 75 SARS-CoV-2 positive individuals recovered completely in 4–5 days after receiving 32 mg lactoferrin (liposomal bovine lactoferrin) daily for ten days with zinc (Chang et al., 2020).

#### 5.3.2 Honey

In honey, water, sugars, enzymes, amino acids, flavonoids, organic acids, phenolic acids, minerals, vitamins, and volatile compounds are all present (da Silva et al., 2016). Honey is mostly sugar (90-95%), with 75 percent monosaccharides (fructose and glucose) and 10 to 15% disaccharides (sucrose, turanose, maltose, maltulose, isomaltose, nigerose, kojibiose, trehalose), and trisaccharides (melezitose and maltotriose) and very minute amounts of other sugars (Belitz et al., 2004). Furthermore, except for glutamine and asparagine, honey contains proteins, comprising essentially free amino acids and enzymes. Proline makes up the majority of the amino acid in honey (Lloyd, 2015).

Honey and its major components are proved effective in treating a variety of viral infections in numerous investigations. Herpes zoster (Hashemipour et al., 2014), rubella (Zeina et al., 1996) influenza (Watanabe et al., 2014), herpes disease (Semprini et al., 2017), respiratory syncytial virus (Zareie, 2011), AIDS (Behbahani, 2014), immunodeficiency virus (Al-Waili et al., 2006), viral hepatitis (Abdulrhman et al., 2016), gingivostomatitis (Awad and Hamad, 2018), rabies rhinoconjunctivitis (Igado et al., 2010), and COVID-19 are among the diseases that honey and its main components can combat (Filipe, 2020). Honey’s antiviral activities and their primary components have a large and unknown method of action. Honey’s antiviral activity is frequently linked to anti-oxidant, anti-inflammatory, anti-resistance, and anti-apoptotic activities through altering cellular signaling pathways such as MAPK, NF-kB, Nrf2, and others (Docherty et al., 2004; Shakibaei et al., 2011a; Shakibaei et al., 2011b; Vickers, 2017; Buhrmann et al., 2019; Buhrmann et al., 2020). Furthermore, these agents have a direct effect on the structure of the virus, such as when honey and its primary components interact with structural and non-structural proteins in the virus, or when they connect to virus target receptors (Yaghoobi and Kazerouni, 2013).

More knowledge of viral replication in the host cell could aid in the development of innovative antiviral medicines that target viral replication while limiting drug resistance. The first stage of viral replication is virus attachment, penetration, and decorating; the second stage is viral genome replication, transcription, and translation; and the final stage is viral assembly. Interruption of the proteins required for viral attachment and entrance into host cells is one mechanism underlying honey’s antiviral effect (Münstedt, 2019). Honey disrupts disulfide bonds in the HA receptors, preventing the influenza virus from attaching to the host cell surface (Watanabe et al., 2014).

Two significant class I fusion protein family members are influenza hemagglutinin protein HA and coronavirus spike protein (Belouzard et al., 2012). The spike protein of SARS-CoV is required for viral interaction to the ACE2 receptor on host cells. Interestingly, chrysin (400 mM) was found to have a weak inhibitory effect on the interaction of S protein with ACE2 (Ho et al., 2007), and both ACE2 and 3CLpro have been identified as antiviral therapeutic targets. Kaempferol and quercetin also had a strong affinity for the SARS-CoV-2 3CL hydrolase (Chen et al., 2012). Kaempferol and quercetin were able to bind to ACE2 and modulate signal pathways such as prostaglandin-endoperoxide synthase 2 (PTGS2), caspase 3, B-cell lymphoma 2 (Bcl-2), and Kaposi’s sarcoma, all of which are linked to herpes virus infection, measles, hepatitis C, human cytomegalovirus, and Epstein–Bar.

Viruses are encoded for the ion-selective channels in the infected cell’s membrane (Fischer et al., 2012; Sauter et al., 2014), and once these channels are activated, the virus is released into the cell and replicates (Schwarz et al., 2012). Ion channel inhibitors can prevent virus generation while allowing the infected cell’s immune system to function more effectively (Schwarz et al., 2014). SARS-CoV encodes for an ion-permeable channel in its open reading frame 3a (ORF3a). Flavonoids have been demonstrated to suppress viral release by activating ion channels. Kaempferol has been proposed as a potential inhibitor of coronavirus 3a-channel proteins (Wang et al., 2014).

Furthermore, chrysin has been shown to suppress viral replication by preventing viral RNA replication and synthesizing viral capsid proteins without causing cytotoxicity. One of the key goals for anti-viral drugs is the regulation of molecular signaling pathways. Kaempferol suppresses the influenza virus replication by activating the opposite cell-autonomous immune responses by altering mitogen-activated protein kinase (MAPK) signaling pathways (Dong et al., 2014). Furthermore, kaempferol and quercetin have decreased SARS-CoV-2 replication by targeting phosphatidylinositol-4,5-bisphosphate 3-kinase catalytic subunit gamma (PIK3CG) and E2F1 (E2F Transcription Factor 1) via the PI3K/Akt signaling pathway. In addition, quercetin and kaempferol reduced SARS-CoV-2 replication via altering the JAK/STAT signaling pathway (Huang et al., 2020).

## 6 Anti-coronavirus metabolites from natural sources

The virus takes precedence over the host’s physiology. In other words, developing an effective medication with fewer side effects is problematic in creating every antiviral medicine, including synthetic medication. In this sense, the pharmaceutical sector frequently moves to natural antivirals (Denaro et al., 2020).

Since the outbreak of SARS in 2003, numerous natural sources of flora and animals have been examined for the efficacy of anti-SARS-CoV-1 or utilized in medication formulation as a scaffold. Some natural constituents include flavones, flavonols, fatty acids, tannins, terpenes, and alkaloids. The diverse mechanisms used by each phytoconstituent category to inactivate coronaviruses account for various such chemical classifications. However, the molecular structures of all these natural molecules share general characteristics that support Jo et al. (2020), who found that SARS-CoV-1 suppression necessitates a molecular structure with a lipophilic aromatic ring, OH groups, and polysaccharide moieties based on in silico research. Even though not all anti-SARS-CoV-1 molecules have a benzene ring, their chemical structures have hydrophobic and lipophilic areas and potentially create numerous H-bonds via OH groups.

Most natural anti-SARS-CoV-1 compounds also have pharmacological effects on all other forms of virus or disease. The Marine sponge mycalamide A is biological against the herpes virus and its homolog, mycalamide B (Donia and Hamann, 2003). Furthermore, myricetin, a flavonol, has antiviral properties against leukemia, HIV, and influenza (Zakaryan et al., 2017). Lycorine is also renowned for its wide range of pharmacological purposes, including antioxidant, antibacterial, antitumoral, anti-inflammatory, and anticancer properties (da Silva Antonio et al., 2020).

**Figure 2:**
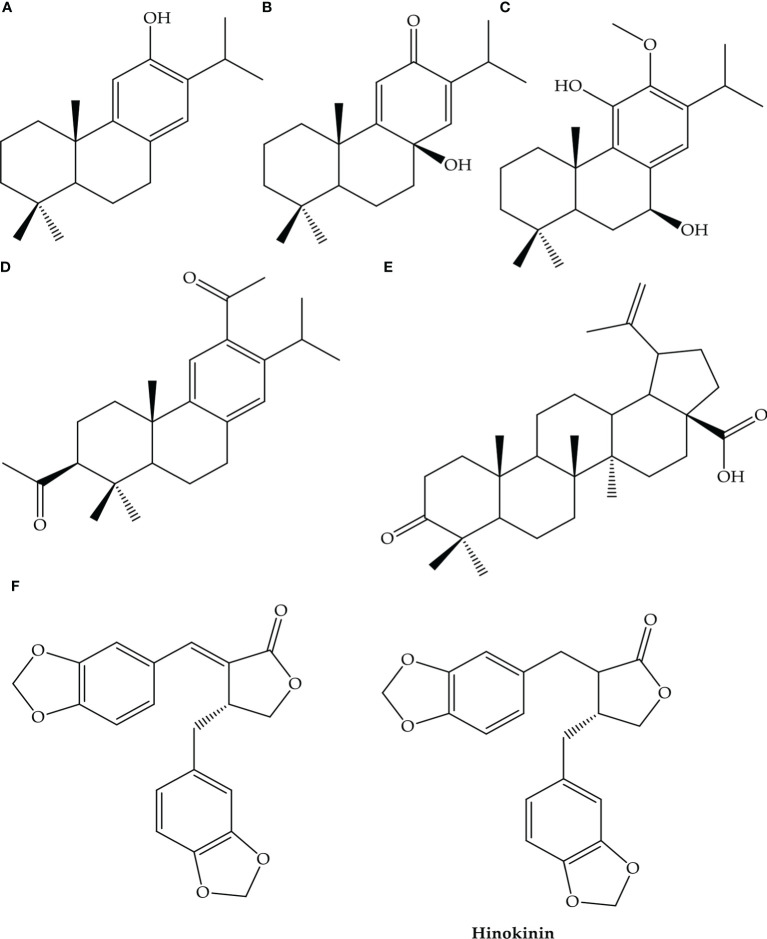
Natural derivatives with antiviral activity against SARS-CoV- 1. Ferruginol **(A)**, 8β-hydroxyabiet-9(11),13-dien-12-one **(B)**, 7β-hydroxydeoxycryptojaponol **(C)**, 3β,12-diacetoxyabiet-6,8,11,13-tetraene **(D)**, betunolic acid **(E)**, and savinin **(F)** (Prasansuklab et al., 2020).

**Figure 3:**
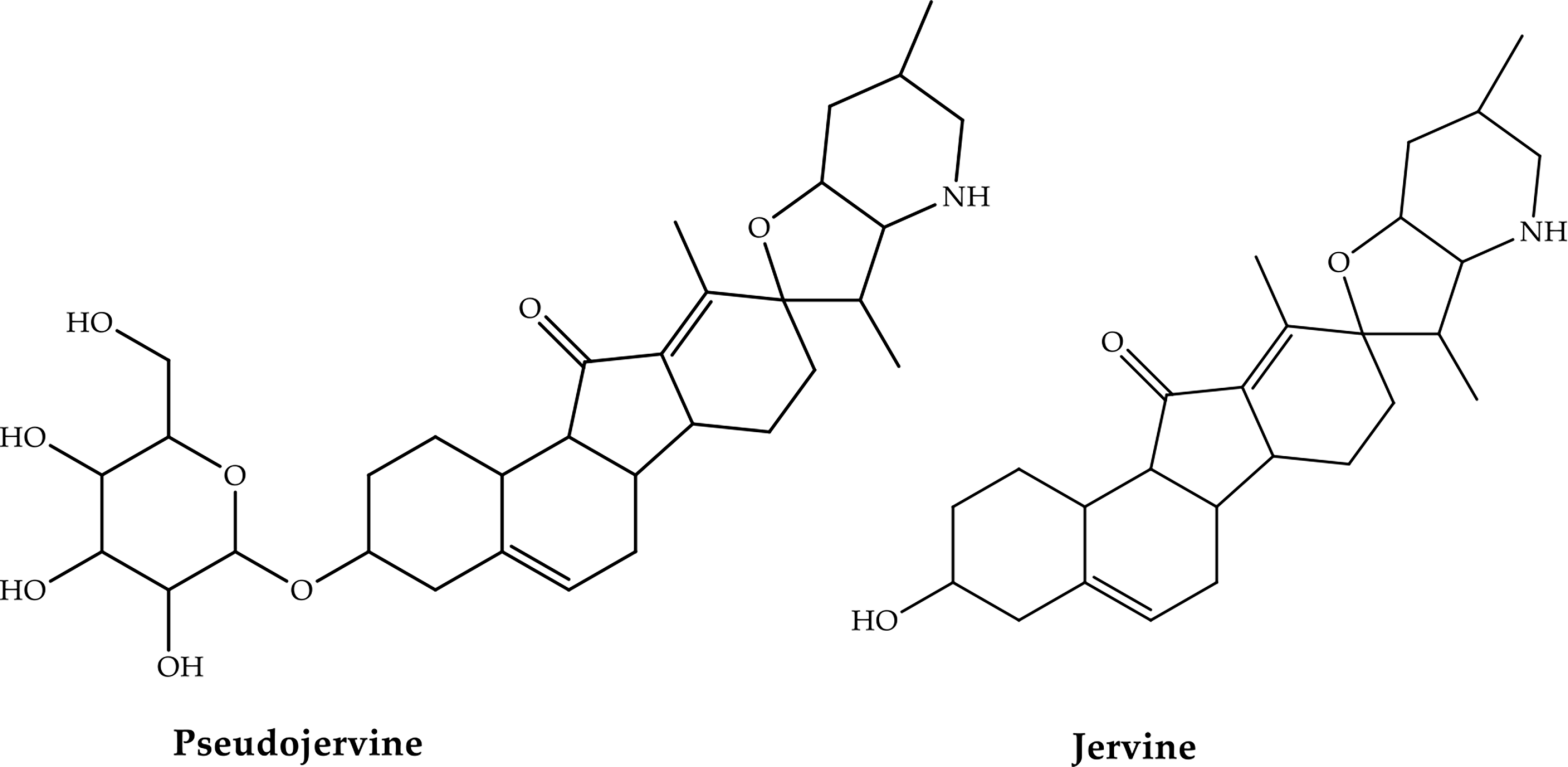
Toxic alkaloids of Veratrum with antiviral activity (Prasansuklab et al., 2020).

The IC50 values ranging from 0.63 (butanaic acid) to 1.57 mM (3 b,12-diacetoxyabiet-7, 8,11,13-tetraene) have potential effects on SARS-CoV-2. Natural metabolites ferruginol (A), 8b-hydroxyabiet-9(11)13-died-11-on(B), 7bhydroxydioxycryptojaponol(C), 3B,12-diacetoxyabiet-6,8,11,13-tetraene and saving(F) have potential effects to SARS-CoV-2 (**Figure 2**) (Prasansuklab et al., 2020). Another toxic alkaloid veratrum treats as ACE2 inhibitors in SARS-CoV-2 (**Figure 3**) (Prasansuklab et al., 2020).

### 6.1 Inhibitors of ACE2

**Figure 4:**
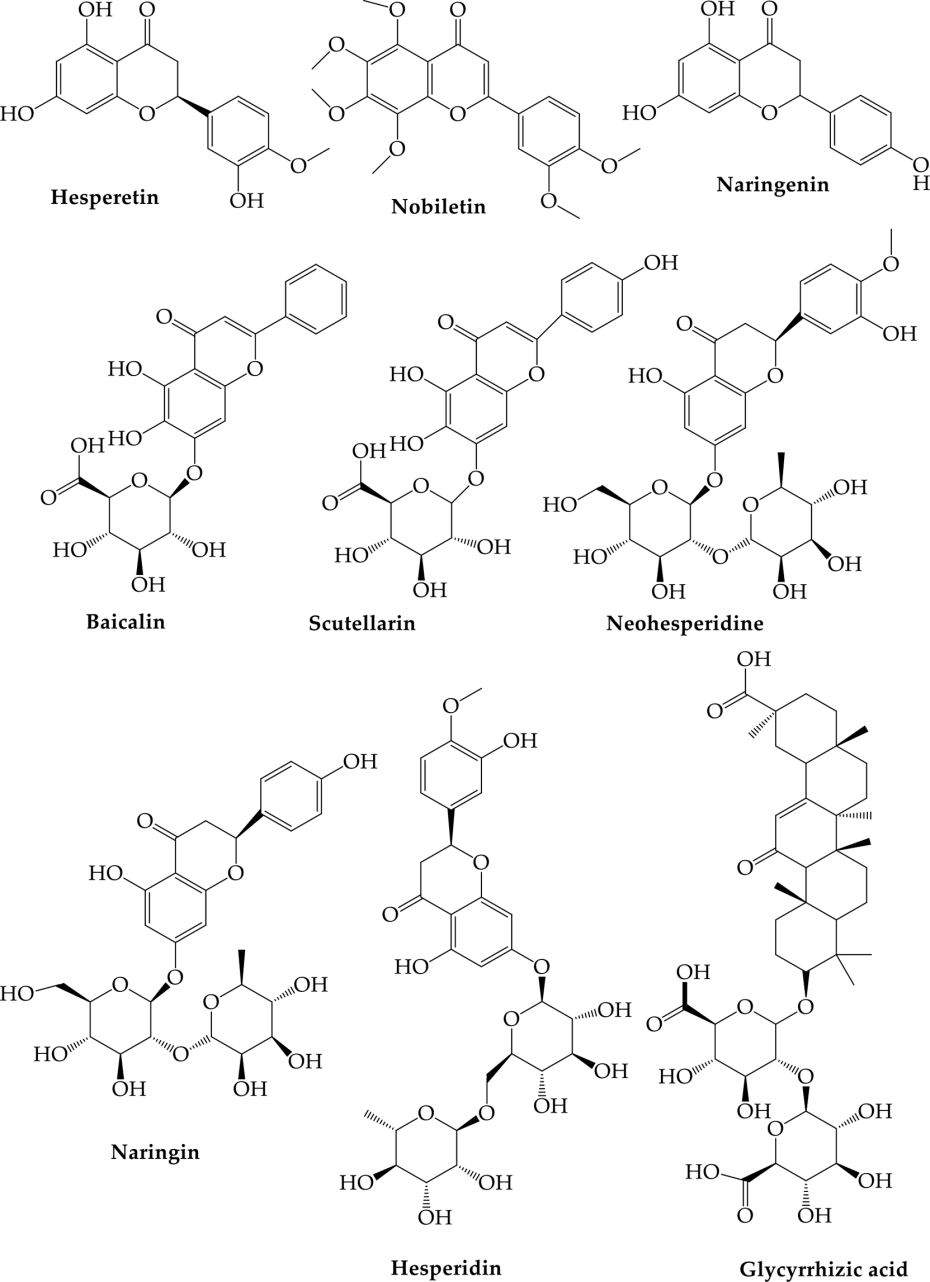
Metabolites virtually screened as ACE2 inhibitors of SARS-CoV-2 (da Silva Antonio et al., 2020a).

When ACE-2 was found as the principal receptor for COVID-19 in humans, researchers focused their efforts on figuring out how to modulate it so that the virus could be controlled. The ACE inhibitory effect is found in many natural compounds widely employed in ethnobotanics and, in some instances, are firmly rooted in human diets (Barbosa-Filho et al., 2006; Daskaya-Dikmen et al., 2017). Bioproducts, including ACE inhibitors, are widely used because synthetic compounds, like enalapril, were created utilizing a scaffold library of natural metabolites. This illustrates their viability as new drug resources; they have fewer adverse effects than synthetic pharmaceuticals, and natural compounds can have lower IC50 values in some circumstances (Daskaya-Dikmen et al., 2017). Flavonoids, xanthones, alkaloids, peptides, terpenes, and tannins are among the phytoconstituents families that riches of ACE inhibitors found in natural goods. Ancient Chinese plants, such as Citrus species, were first scanned in silico for natural anti-COVID-19 chemicals (Meneguzzo et al., 2020). Eleven non-synthetic compounds with antiviral activity were discovered after artificial docking studies found that plants native to China intermediate with ACE-2 against COVID-19 (Meneguzzo et al., 2020). The natural substances suggested in traditional Chinese medicine are botanical line (baicalein-7-O-glucuronide), scutellarine (scutellarein-7-glucuronide), hesperetin, nicotianamine, naryingine, hesperidin, and nobiletin (**Figure 4**) (Meneguzzo et al., 2020), (Cheng et al., 2020; da Silva Antonio et al., 2020).

**Figure 5:**
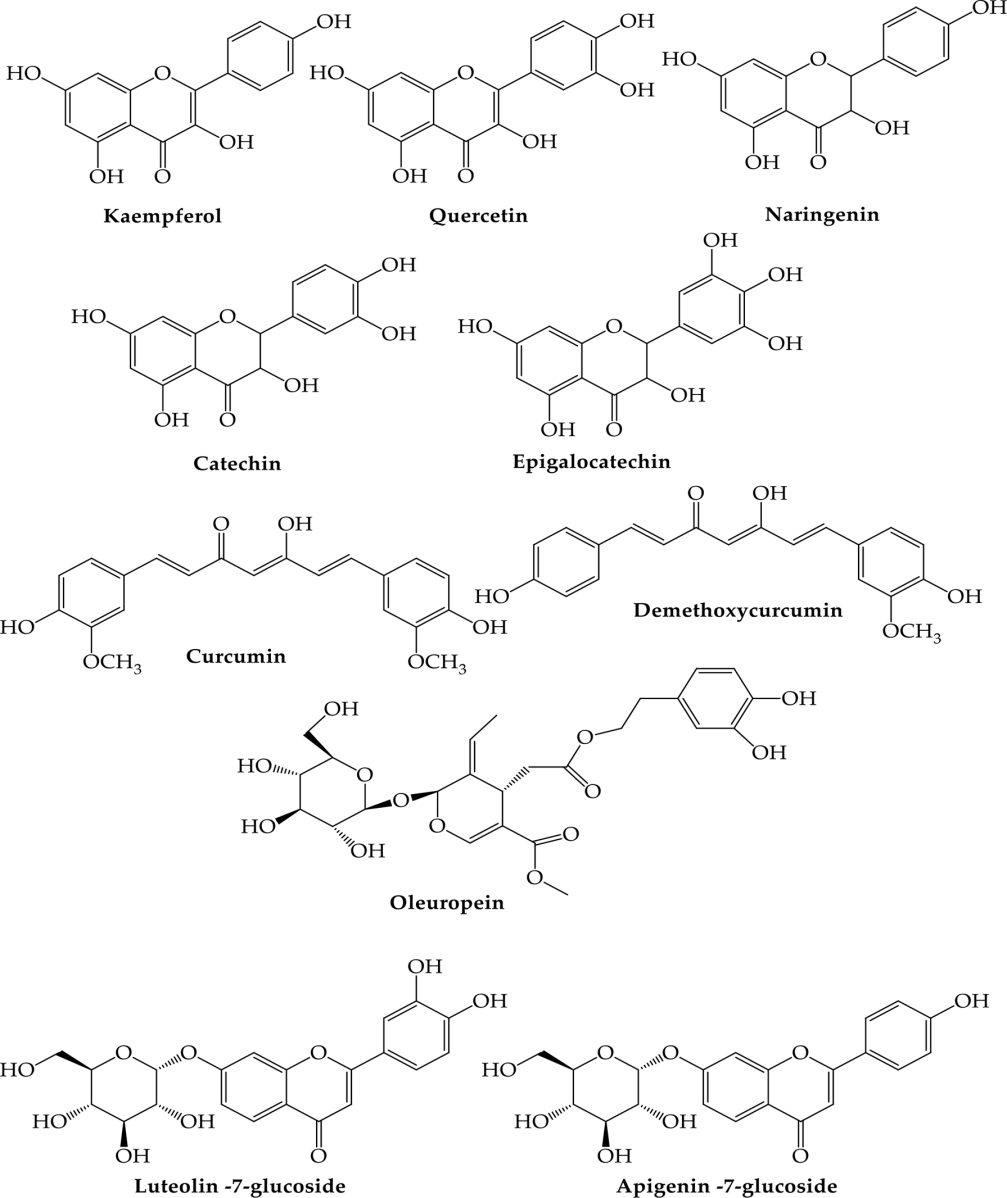
Natural metabolites are suggested as inhibitors of the 3CL^pro^ of the SARS-CoV-2 (da Silva Antonio et al., 2020a).

The chromatographic fingerprints which follow the molecular docking process of a Chinese natural herb used to treat COVID-19 have also identified Veratrum nigrum L. preparations and alkaloids as SARS-CoV-2 ACE2 inhibitors (Cheng et al., 2020). The majority of the proposed alkaloids, such as hupehemonside, pseudojervine, and imperial, are Liliaceae-specific. Thus, Veratrum species are widely recognized as dangerous when ingested after boiling because of the presence of a certain set of alkaloids that are either important or contain minor chemicals in their chemical composition (da Silva Antonio et al., 2020a). A counterpart of jervine (**Figure 5**), a poisonous alkaloid, is among the anti-SARS-CoV-2 alkaloids discovered using molecular docking. Consequently, although the presence of various anti-COVID-19 alkaloids, Veratrum species must be used with caution.

### 6.2 3CLpro Inhibitors

Scientists are paying more and more attention to the suppression of 3CLpro, the major protein of COVID-19, because it could impede virus implantation on the organism (Cui et al., 2020; Gentile et al., 2020; Gurung et al., 2020). Although the enzyme 3CLpro is viral-specific, it has several structural similarities with those discovered in SARS-CoV-2, similar to the one found in SARS-CoV-1 (96.08 percent) (Gurung et al., 2020). In silico study by Gurung et al. (2020) )revealed that the terpenoids bonducellpin D and causal in B, as well as the flavonoid 5,7-dimethoxy flavanone-4′-O—d-glucopyranoside, have affinities for 3CLpro of SARS-CoV-1, SARS-CoV-2, & MERS-CoV varying from a range of -8 to -11 kilocalorie mol-1. Even though the terpenoids and flavonoids discovered by great scientist Gurung and colleagues are found in natural medicinal plants (Caesalpinia minax) and European mistletoe (Viscum album), the concurrent suppression of many SARS-CoV-2 strains offers hope for developing a COVID-19 treatment.

Khaerunnisa et al. evaluated (Khaerunnisa et al., 2020) Kaempferol, quercetin, luteolin- 7-glucoside, dimethoxy curcumin, naringenin, apigenin-7-glucoside, oleuropein, catechin, curcumin, and epigallocatechin which are the most needed derivatives in the molecular docking industry employed to search for the 3Cpro of SARS-CoV-2 (da Silva Antonio et al., 2020a; Joshi et al., 2020; Rane et al., 2020). Those flavonoids’ anti-SARS-CoV-2 effect is favorable because they are widely dispersed in angiosperm botanical groups such as Lauraceae, Lamiaceae, Apiaceae, and Leguminosae (Gentile et al., 2020).

## 7 Prospects of using computational techniques to screen possible anti-COVID-19 agents from plants

Zhang et al. have recently reported the crystal structure of the main protease SARS-CoV-2 (Mopar, sometimes referred to as 3CLpro) necessary for viral replication (Monteil et al., 2020). The crystal structure enables the screening of potential lead molecules active against COVID-19 by using computer analysis of SARS-CoV proteases and other similar components. A molecular docking study includes kaempferol (73), quercetin (22), luteolin-7-O-glucoside (74), desethoxy curcumin (75), desmethhoxy calcumin (76) and curcumin (72) [105]. SARS-CoV-2 host receptor, ACE2, is the same as SARS-CoV host receptor, according to prior study; hence SARS-CoV-ACE2 inhibitors may also be able to obstruct the SARS-CoV-2 receptor. The possible inhibitors were discovered as baicalin (81), scutelarin (82), hesperetin (38), nicotinamide (83), and glycyrrhizin (26), and could be used as anti-2019-nCoV drugs based upon Chen and Du molecular docking (Zhang et al., 2020c). Molecular docking is a quick way to find potential active compounds during the creation of medications. It can be used to characterize molecular interactions and binding affinities. In vitro or in vivo antiviral investigations must instead support molecular docking data depicting a medicine that is tangible in its activity. Research has shown that the positive link between docking scores and pharmacological activity is weak, and inactive docking drugs are ineffective (Costanzi and Vilar, 2012). This emphasizes the need for wet laboratory tests to verify biological activity, particularly in a global pandemic.

## 8 Potential leads from southern African plants

In a considerable part of the population of southern Africa, traditional medicine represents the principal source of health treatment. Traditional knowledge systems record the use of a plant to cure a specific symptom instead of a particular illness or infectious organism. Southern African plants historically used to treat cough, fevers, colds, and flu are listed in this section as potential candidates for SARS-CoV-2 testing and associated targets.

The ability of Artemisia afra to control coronaviruses has not been evaluated. A. annua, on the other hand, reduced viral replication of SARS-CoV BJ-001 with an EC50 of 34.5 2.6 g/mL in Vero cells. Although these two separate species exist, many chemical substances are retained in Artemisia. Nonetheless, the little chemical nuances and profile have a considerable impact on biological activity (Zibaei and Firoozeh, 2013). Closely related medicinal plant species may also produce comparable or chemically similar chemicals responsible for their physical action (Sharma et al., 2019). This is the fundamental definition of chemotaxonomy: “closely related plants have the same or comparable chemical profiles” (Hao et al., 2020). Many characteristics of the Cissampelos genus, for example, were explored in a review study, including ethnobotanical elements, extracted phytochemicals, and biological activity of the various species. The presence of alkaloids is thought to be responsible for most of the physical activity observed in Cissampelos species. Furthermore, the paper discusses the presence of comparable chemicals in different Cissampelos species. Many biological functions, including clinical study outcomes, are linked to the waistline, and this molecule was isolated from Cissampelos ovalifolia and Cissampelos sympodial (da Silva Mendes et al., 2020). Another example is the Ziziphus mucronata plant in Southern Africa, which is not researched in antiviral activity. However, cyclopeptide alkaloids produced from Z. jujuba have suppressed the PEDV of Vero cells with SI values ranging between 7,98 and 47,11. (Liu et al., 2015)

MERS-CoV 3CLpro was suppressed by helichrysetin, a chemical present in many Helichrysum species (Kim et al., 2019b). EPs® 7630, a commercial product of Pelargonium salides, was shown to offer a weak selectivity index of 2.3 when assessed in caco-2 against the human coronavirus strain 229E. Two main compounds found for their anti-coronavirus potential were investigated in traditionally used plants. Sitosternol EC50 was 1210 M against human coronavirus and detected in Dodonaea viscosa and Prunus africana (HCoV-NL63) (Lin et al., 2005). More testing of the mentioned plant species could lead to identifying a lead candidate for the treatment of COVID-1 SARS-CoV viral Replication was decreased by EC50 by 3.4 M, CC50 by 2.5 M, and SI by 7,3 by Reserpine, the primary component for Rauvolfia caffra [118]. More tests of the species could lead to a lead candidate for the COVID-19 treatment being identified.

In vitro, in vivo, and clinical trials of herbal medicine on African medicinal plants were all considered. Studies on medicinal plants in Africa were eligible for inclusion if they were undertaken to determine antiviral activity using available scientific methods. African medicinal herbs that have shown antiviral activity and could be possibilities for COVID-19 treatment (Beressa et al., 2021; Gyebi et al., 2021). An in silico screening of 62 alkaloids and 100 terpenoids from African medicinal plants was carried out against coronavirus 3-chymotrypsin-like protease (3CLpro), a well-defined hit-list of seven compounds. In addition, four nontoxic, druggable plant-derived alkaloids and terpenoids that bind to the SARS-CoV-2 3CLpro receptor-binding site and catalytic dyad were discovered. More than half of the top 20 alkaloids and terpenoids demonstrated a binding affinity for the SARS-coronavirus 3CLpro that was higher than that of reference inhibitors. Bisnorterpenes’ 6-oxoisoiguesterin showed the maximum binding affinity to the 3CLpro of SARS-CoV-2, whereas Bisnorterpenes’ 20-epi-isoiguesterinol, Bisnorterpenes’ isoiguesterin, and Cogniauxia podolaena’s 20-epibryonolic acid were the top docked molecules to the 3CLpro of SARS-Co Natural agents from the alkaloids and terpenoids classes are capable of inhibiting the 3CLpro with a strong inhibitory pattern to both SARS-CoV-2 and SARS-CoV, according to the study (Gyebi et al., 2021).

## 9 Anti-corona viral natural products with unidentified modes of action

NormalEven though their activity instruments have not yet been set up, some normally active substances have been displayed to have a beneficial outcome in repressing SARS-CoV. Subsequently, the synthetics found in earlier examinations may likewise forestall COVID-19 contamination. Three Cinnamomi anthocyanins, cinnamtannine B1, procyanidin A2, procyanidin B1, and both HIV/SARS-CoV S and SARS-CoV wildlife have been shown to exhibit inhibitive actions. However, clathrin-operated endocytosis does not occur. The impacts of sure rough plant separates were likewise contemplated, and it was found that a fluid concentrate of Caryophylli Flos showed impressive concealment of pseudovirus (IC50 14 58.8 mM) and wild-type infection (IC50 14 50.1 mM) (Zhuang et al., 2009). Besides, lycorine, a characteristic alkaloid from Lycoris emanate, has been proposed as an enemy of SARS-CoV particles with an IC50 of 15.7 nM (Li et al., 2005; Prasansuklab et al., 2020).

## 10 Traditional Chinese herbal medicine against COVID-19

Traditional Chinese medicines (TCM) have a long history, preventing and treating several epidemic diseases. TCM’s assistance during the SARS outbreak in 2003 also had a significant therapeutic effect. Over 3100 TCM medical personnel were dispatched to Hubei province during the COVID-19 treatment phase. The TCM scheme was incorporated into the COVID-19 diagnosis and treatment protocol, and TCM professionals were fully involved in the entire rescue process (Lu et al., 2020; Ren et al., 2020). TCM decoctions, Chinese patent medicine, acupuncture, and other TCM-specific therapies were widely used, and the majority of cases were treated based on syndrome distinction. TCM wards were developed, alongside a specialized hospital; additionally, the TCM staff participatein treatment as a group. The overall number of confirmed TCM cases presently stands at 60,107. In 102 cases of mild TCM-treated symptoms, the period for symptom disappearance was shortened by two days. The body’s recovery temperature was shortened by one day, hospital stay was shortened by 2.2 days, CT’s improvement rate was increased by 22%, and clinical cure rate was increased by 33%. The rate of infection decreased by 27.4%.

Furthermore, the average duration of hospital stays and the time it took for nucleic acid to turn negative was decreased by over two days when severe patients were treated with TCM. Based on current therapy discoveries, TCM offered to make effective prescriptions, such as Qingfey Pay Decoction (QPD), sheganmahuang decoction, and qingfei touxie fuzheng recet, etc. Based on current therapeutic findings. This kind of QPD is Ephedra Herba, Glycyrrhizae Radix, Rhizoma Praepropata cum Melle, Gipsum Fibrosum, Alismatis Rhizoma, Polyporus, Atractylodis, Macrocephalae, Poriya, Bupleuri, Scutellum, Gypsum Fibrosum, and Cinnamomi Ramulus. Of the 701 QPD cases validated, 130 were cured and discharged, with 51 instances losing clinical signs, 268 improving, and 212 remain stable (Ren et al., 2020). QPD has a successful cure rate of more than 90% against COVID-19. According to TCM theory, COVID-19’s target organ is the lung, and the etiology is “damp and toxic plague.” QPD has a multi-component and multi-target regulatory impact, according to a network pharmacology study.

As 16 plants correspond to the lung meridian, the decoction is generally used to treat lung disorders. Furthermore, rising and falling with the spleen and stomach can dehumidify the body, and it has been demonstrated to protect the heart, kidneys, and other organs. The bulk of the prospective targets were found to be co-expressed with ACE-2, COVID-19’s receptor, implying that COVID-19 could be improved. By interacting with several ribosomal proteins, it can prevent COVID-19 from reproducing. COVID-19 can generate an inflammatory storm as well as a strong immunological response (Xu et al.). A functional enrichment study showed that QPD might manage and ease excessive immune reactions by modulating immunological and cytokine pathways and eliminating inflammation (Zhao et al., 2020). Furthermore, utilizing molecular docking predictions, it was observed that the formula’s patchouli alcohol, ergosterol, and shionone had a better anti-COVID-19 activity, resulting in new molecule structures for therapeutic development (Ren et al., 2020; Wu et al., 2020b).

There are more than 18 recommended TCMs to prevent and treat COVID-19, according to the officially issued 7th and 8th trial versions of Diagnosis and Treatment Protocol for COVID-19 in China and other references (Lee et al., 2020; Li et al., 2020b; Luo et al., 2020b), there are more than 18 recommended TCMs to prevent and treat COVID-19, covering the medical observation period (suspected cases) to the clinical treatment period (confirmed cases), including distinct disease stages of mild, moderate, severe, and critical. Jinhua Qinggan granules, Lianhua Qingwen capsule (granules), and Xuebijing injection are three highly recommended Chinese patent medicines (CPMs), and Qingfei Paidu decoction, Huashi Baidu formula, and Xuanfei Baidu formula are three Chinese medicine formulas with proven efficacies in treating COVID-19 (Li et al., 2020a; Wang and Qi., 2020b). The granules of Jinhua Qinggan clear heat, cleanse, and diffuse the lungs. It consists of 12 herbal medications derived from the Maxingshigan Yinqiaosan formula, which may have reduced the time it took for patients to recover from the H1N1 influenza virus infection in 2009 (Wang et al., 2011). In 2003 (Xiao et al., 2020; Hu et al., 2021), a unique CPM for the treatment of SARS was Lianhua Qingwen capsule (granules), which contained 13 herbal medicines and had a clinical indication for eliminating heat, diffusing the lung, and detoxifying. Xuebijing injection, a five-herbal injection medicine with a therapeutic indication for dissolving stasis and detoxifying, was produced and commercialized during SARS and was derived from a modified Xuefu Zhuyu decoction. Qingfei Paidu decoction is a Chinese medicine formula that combines 21 herbal remedies from five classic formulas from the Treatise on Febrile Diseases. It is the first suggested universal therapy solution for all stages of COVID-19 (Li et al., 2020a; Wang et al., 2021), clearing the lungs and calming panting. The Huashi Baidu recipe is made up of 14 different medicinal plants. It is used to eliminate heat, detoxify, and remove dampness, and is mostly used to treat mild, moderate, and severe COVID-19 patients (Xiong et al., 2020; Shi et al., 2021). The 13 therapeutic plants in the Xuanfei Baidu recipe are drawn from ancient formulations such as Maxing Shigan decoction and Maxing Yigan decoction. It detoxifies and removes blood stasis, diffuses the lungs, eliminates moisture, and clears heat, and is primarily used to treat mild to moderate COVID-19 patients (Chen et al., 2020a). In comparison to orally administered TCMs (Song et al., 2020; Zhang et al., 2020b; Lyu et al., 2021), Chinese herbal injections, such as Xiyanping injection, Reduning injection, Tanreqing injection, Shenfu injection, Shengmai injection, were more suitable as supplemental treatments for severe or critical COVID-19 cases because of their advantages of fast absorption, high bioavailability, and clearer ingredients (Lyu et al., 2021).

## 11 Impact of marine natural products in COVID-19

Bioactive substances found in marine algae include vitamin E, B12, phycocyanin, lutein, and polysaccharides (Herrera-Calderon et al., 2020). Lambda carrageenan is a polysaccharide derived from marine red algae that contains antiviral, antibacterial, anti-cancer, and anti-coagulant effects. It has been shown to efficiently inhibit both the influenza virus and SARS-CoV-2. A study discovered that the marine polysaccharide repressed viral replication and lowered viral protein expression in a dose-dependent way (Akter et al., 2021). As the lambda-carrageenan dose was increased from 0 to 300 g/mL, the presence of spike viral proteins on SARS-CoV-2 and influenza A viral proteins dropped drastically (Zahran et al., 2020). Inhibition of influenza virus and SARS-CoV-2 had EC50 values of 0.3–1.4 g/mL and 0.9–1.1 g/mL, respectively. There was no evidence of host cell toxicity at dosages up to 300 g/mL. Mice given lambda-carrageenan after being infected with the SARS-CoV-2 virus had a 60% survival rate, indicating that the polysaccharide inhibited viral entry and reproduction. These results show that lambda-antiviral carrageenan has antiviral properties, giving it a viable COVID-19 therapeutic option. While these findings are encouraging, it is important to keep in mind that lambda-carrageenan may have undesirable side effects. Previous study has indicated that oligosaccharides coming from the carrageenan family (kappa and lambda-carrageenan) can inhibit the formation of new blood vessels, causing blood vessel development to be impaired. At 200 g/mL, they were also found to inhibit human umbilical vein endothelial cell migration, proliferation, and tube formation. These data show that there could be harmful effects in humans, but additional in vitro and in vivo toxicology study is needed. These findings must be considered when developing lambda-carrageenan as a SARS-CoV-2 inhibitor.

Scleractinia-associated organisms like bacteria and fungus have therapeutic promise as well (Abdelmohsen et al., 2017; El-Hossary et al., 2017; Shady et al., 2017; El-Hossary et al., 2020; Zahran et al., 2020). Because they produce a variety of metabolites, these organisms have been related to inflammation and viral infection (El-Hossary et al., 2020; Zahran et al., 2020). Scleractinia-related metabolites were investigated, and molecular docking was utilized to uncover SARS-possible CoV-2’s antiviral effects. Terphenyllin and Tirandamycin A, two particular microbial metabolites, have been identified to form hydrogen bonds with the main protease (Mpro) and dock with high affinity (Zahran et al., 2020). These marine metabolites are thought to be promising candidates for blocking the virus’s main protease, which is essential to the virus’s survival. Seventeen potential Mpro inhibitors were found in the class phlorotannins isolated from Sargassum spinuligerum brown algae in a similar study. The compounds have significant hydrogen bonding and hydrophobic interactions with Mpro, with docking energies ranging from 14.6 to 10.7 kcal/mol. The SARS-CoV-2 RNA polymerase and nsp7/8 are also required for RNA replication and viral protein production. Remdesivir is a well-known RNA polymerase inhibitor, and three Scleractinia metabolites have been found to bind the polymerase in the same place as remdesivir. This data suggests that by reducing viral replication, these marine metabolites could be effective in the treatment of COVID-19. In addition, a study on Mpro utilizing molecular docking found that a number of marine compounds have possible binding interactions (Khan et al.).

## 12 Genome Replication Inhibition

Two viral protease enzymes are necessary for SARS-CoV replication. 3CLpro and 3PLpro are two viral protease enzymes (PLpro). Helicase and RdRp are two additional enzymes needed to replicate SARS-CoV. As a result, these inhibitors can be examined as a therapeutic possibility in COVID-19 treatments (Boozari and Hosseinzadeh, 2021).

### 12.1 Papain-like Protease Inhibitors

Another CoV protease enzyme, papain-like protease (PLpro), is implicated in proteolysis, innate immunity antagonism, deubiquitination, and viral multiplication (Clementz et al., 2010), making it a valuable target for antiviral medicines. Tanshinones isolated from Salvia miltiorrhiza with an abietane diterpene structure is among the most promising PLpro inhibiting substances. Tanshinone inhibits both 3CLpro and PLpro. Their activity against PLpro, on the other hand, was noticeably higher than that against 3CLpro. The IC50 values for cryptotanshinone, tanshinone IIA, and dihydrotanshinone I was 0.8, 1.6, and 4.9 M, respectively. Diarylheptanoids, such as Hirsutenone from Alnus japonica (IC50: 4.1 M) and curcumin from Curcuma longa, have been demonstrated to inhibit PLpro (IC550: 4.1 M) (IC50: 5.7 M) (Park et al., 2012a). Prenylated chalcones isolated from Angelica keiskei, such as xanthoangelol E and xanthoangelol F, inhibit PLpro noncompetitively with IC50 values of 1.2 and 5.6 M, respectively (Park et al., 2016).

### 12.2 RNA-dependent RNA Polymerase Inhibitors

RdRp is required for positive-strand Ribonucleic acid (RNA) virus replication and transcription (Chan et al., 2015). Antiviral medicines that block RNA polymerase are a suitable choice for coronavirus therapy. The literature says that the biflavonoid skeleton is a potential inhibitor of RdRp, with the most promising of all amentoflavone and robustaflavone. Dacrydium araucarioides produced a bioflavonoid structure of the sotetsuflavone. In vitro tests showed that sotetsuflavone, an IC50 of 0.16 M, is a potent inhibitor of the RdRp Dengue virus. According to SAR investigations, the C30-C600 link is critical for inhibitory activity. The amount and location of methylation groups also influenced movements (Coulerie et al., 2013).

## 13 Clinical studies

Phytochemical based formulation has provided promising and adequate results in treating COVID-19 and in recruiting or other stages. These formulations will be potential in developing the novel drug for COVID-19 because they have some promising anti-viral activities. Several numbers of formulations and agents are as follows: (**Table 2**) (Wang et al., 2020a; Alam et al., 2021).

**Table 2:** Ongoing clinical trials of several plants, functional foods, and plant-based products against SARS-CoV-2 infection.

No	Products	Phase	Participant Numbers	Intervention	Results	Clinical Trails. Gov Identifier	References
1	Fuzheng Huayu Tablet	Phase II	160 participants	0.4 g/tablet, 1.6 g/time, 3 times/day	The fraction of people with pulmonary fibrosis who are improving, and blood oxygen levels Clinical, Saturation	NCT04279197	(Wang et al., 2020a; Alam et al., 2021)
2	Natural honey	Phase III	1,000 participants	1 gm/kg/day for 14 days, divided into 2–3 doses	The rate of recovery from positive to negative swaps and the time it takes for a fever to return to normal are all factors to consider	NCT04323345	(Wang et al., 2020a; Alam et al., 2021)
3	Anluohuaxian	Not applicable	750 participants	6 gms twice a day	Changes in lung high-resolution computer tomography, Change in walking distance over 6 minutes, Changes in the lung’s essential capacity	NCT04334265	(Wang et al., 2020a; Alam et al., 2021)
4	Escin	Phase II, Phase III	120 participants	12 days of oral administration of conventional treatment and Escin tablet (40 mg thrice a day)	The differences in oxygen intake methods, the length of hospitalization (days), the length of stay in critical care units, and pulmonary function tests were all used to determine the death rate	NCT04322344	(Wang et al., 2020a; Alam et al., 2021)
5	*Caesalpinia spinosa* (Molina) Kuntze (Fabaceae)	Phase II, Phase III	100 participants	Every 12 hours for 14 days, take a P2Et active extract capsule equivalent to 250 mg of P2Et + Standard care	The effectiveness of P2Et in reducing the length of a patient’s stay in the hospital. based on clinical suspicion a confirmed instance of COVID-19	NCT04410510	(Wang et al., 2020a; Alam et al., 2021)
6	*Nigella sativa* L. (Ranunculaceae)	Phase II	200 participants	*Nigella sativa* Black seed oil in 500 mg capsules	Determination of the proportion of patients who have recovered clinically, normalization of the chest radiograph, and the rate of problems	NCT04401202	(Wang et al., 2020a; Alam et al., 2021)
7	Essential oil	Not applicable	65 participants	Essential oil Blend 5 drops on a tester strip	At 15 minutes, the State-Trait Anxiety Scale (STAI-S) was determined	NCT04495842	(Wang et al., 2020a; Alam et al., 2021)
8	Plant polyphenol	Phase II	200 participants	Plant polyphenols and placebo are introduced separately, as well as vitamin D3 (10,000 IU)	At 21 days after enrolment, the rate of hospitalization has decreased	NCT04400890	(Wang et al., 2020a; Alam et al., 2021)
9	Silymarin	Phase III	50 participants	420 mg/day, divided into three doses	Time to clinical improvement, clinical outcome, mechanical ventilation duration, hospitalization, and virologic response are all factors to consider	NCT04394208	(Wang et al., 2020a; Alam et al., 2021)
10	ArtemiC (curcumin, artemisinin, vitamin C, and *Boswellia serrata*)	Phase II	50 participants	For the first two days of treatment, ArtemiC will be sprayed orally twice a day	Time to positive clinical improvement, Time to negative clinical improvement PCR for COVID-19	NCT04382040	(Wang et al., 2020a; Alam et al., 2021)
11	Medicinal cannabis (*Cannabis sativa* L., Cannabaceae)	Phase II	200,000 participants	Cannabis, medical	COVID-19 treatment and symptom management	NCT03944447	(Wang et al., 2020a; Alam et al., 2021)
12	Jing-Guan- Fang (JGF)	Not applicable	300 participants	Jing-Guan-Fang (JGF)	The number of COVID- 19 patients after this preventive treatment	NCT04388644	(Wang et al., 2020a; Alam et al., 2021)
13	Licorice extract	Not applicable	70 participants	For ten days, take 250 mg of standardized licorice extract (25% glycyrrhizin, 62.5 mg)	The number of persons recovering from COVID-19 has increased	NCT04487964	(Wang et al., 2020a; Alam et al., 2021)
14	Iota-Carrageenan	Phase IV	400 participants	Four times a day, an Iota-Carrageenan nasal spray or a placebo	Progression to a more advanced level a severe illness condition, defined as a requirement for Long-term oxygen therapy of disease, the frequency of COVID-19 is a virus that causes sickness. beginning within the first week following therapy	NCT04521322	(Wang et al., 2020a; Alam et al., 2021)
15	Acai palm berry extract (*Euterpe* *oleracea Mart.* from Arecaceae family)	Phase II	480 participants	Take one Açaí Palm Berry capsule (520 mg) every 8 hours for a total of three capsules every day for 30 days	Need for mechanical ventilation, hospitalization, and a 7-point ordinal symptom scale	NCT04404218	(Wang et al., 2020a; Alam et al., 2021)
16	QuadraMune™ (Composed of four natural ingredients)	Not applicable	500 participants	For 12 weeks, take two QuadraMune (TM) capsules every day	Prevention of COVID-19	NCT04421391	(Wang et al., 2020a; Alam et al., 2021)
17	Phenolic monoterpenes + colchicine	Phase II	200 participants	In patients with COVID-19 infection, colchicine and phenolic monoterpenes were given to usual treatment	In comparison to the control group, improvements in clinical, radiological, and laboratory symptoms will be assessed in the treated group	NCT04392141	(Wang et al., 2020a; Alam et al., 2021)
18	Cannabidol	Phase I, Phase II	400 participants	Cannabidiol (150 mg twice daily) was given orally for 14 days	Cannabidol’s effect on the cytokine profile in severe and seriously COVID-19-infected patients, as well as its safety and efficacy profile	NCT04731116	(Wang et al., 2020a; Alam et al., 2021)
19	Resistant Starch	Phase II, Phase III	1,500 participants	Nonresistant starch was used in the same amount as the placebo for 14 days in a twice-daily regimen	Calculating the hospitalization rate for COVID-19-related complications	NCT04342689	(Wang et al., 2020a; Alam et al., 2021)
20	Colchicine	Phase III	102 participants	A preliminary dose of 1.5 mg was given, followed by 0.5 mg twice daily for the next 7 days, and then 0.5 mg once daily until the 14-day treatment was completed	The 7-point ordinal scale is used to assess changes in the clinical state of patients The expert panel for the WHO R&D Blueprint, as well as IL-6 concentrations	NCT04667780	(Wang et al., 2020a; Alam et al., 2021)
		Phase II	70 participants	On day 1, a 1.2 mg first dose was given, followed by a 0.6 mg dose after 2 hours. Following that, 0.6 mg in two doses until the 14th day	Evaluation of the risk of developing ARDS, which necessitates upraised oxygen demands, mechanical ventilation, and mortality	NCT04363437	
21	Hesperidin	Phase II	216 participants	In the evening and before bedtime, take 0.5 gm hesperidin capsules with water	Counting the number of people that have COVID-19 symptoms	NCT04715932	(Wang et al., 2020a; Alam et al., 2021)
22	Resveratrol + Zinc	Phase II	60 participants	2 grams resveratrol twice a day + 50 mg zinc picolinate three times a day for five days	Evaluation of COVID-19 viral load decrease and severity	NCT04542993	(Wang et al., 2020a; Alam et al., 2021)
23	Melatonin	Phase II	30 participants	For 14 days, take ten milligrams three times a day	The cumulative incidence of treatment-emergent adverse events must be determined	NCT04474483	(Wang et al., 2020a; Alam et al., 2021)
		Not applicable	55 participants	Melatonin intake of nine milligrams for seven to ten nights	Identification of immune system modulation	NCT04409522	
		Phase II	18 participants	The maximum daily dose is 500 mg	The effect of melatonin on mortality and length of stay in the hospital is being investigated	NCT04568863	
		Not applicable Phase II, Phase III	150 participants 450 participants	10 mg before bedtime for 12 weeks, take 2 mg of melatonin with a lengthy half-life before night	The severity of symptoms can be tracked electronically Melatonin’s usefulness as a prophylactic agent is being investigated	NCT04530539 NCT04353128	(Wang et al., 2020a; Alam et al., 2021)

## 14 Computational studies

Currently, computational tools have been considered the most popular tools for designing and discovering new therapeutic compounds (Ou-Yang et al., 2012; Sliwoski et al., 2014). Since these mentioned compounds have been reported to have strong antiviral activity by renowned scientists in the literature. We have decided to complete an in silico investigation to verify the antiviral effect, and to discover whether these compounds have any harmful effects in the body or are safe, and whether the compounds are safe for aquatic and non-aquatic environments or have adverse effects on the environment.

### 14.1 Molecular Docking Against SARS-CoV-2 Main Protease

A molecular docking study has been employed with the help of Pyrx virtual tools (Dallakyan and Olson, 2015). Before completing the docking procedure, the crystal structure of SARS-CoV-2 Main protease was downloaded from the Protein Data Bank (https://www.rcsb.org/) and the excess ligand and water molecules were cleaned up using Pymol (version 1.3) (DeLano, 2002).

The initial reason for the docking result is hydrogen bonding and hydrophobic bonding, and the standard binding affinity for an effective medication is -6.00 (Cosconati et al., 2010; Nath et al., 2020; Nath et al., 2021a; Sarkar et al., 2021). The reported compounds have been displayed on **Table 3**. It has been seen that all the compounds are highly active against the SARS-CoV-2 main protease and exceed the standard Molnupiravir’s binding energy. At a same time, it has been observed that L.05 (-11.4 kcal/mol), L14 (-10.8 kcal/mol), and L.17 (-10.1 kcal/mol) and L18 (-10.0 kcal/mol) provided the highest activity while using the standard Molnupiravir.

**Table 3:** Binding energy SARS-CoV-2 main protease.

S/N	PubChem ID	Compounds Name	Binding energy SARS-CoV-2 Main proteas(PDB ID: 7MBG) kcal/mol
L01	637394	Hirsutenone	-6.7
L02	10022050	Xanthoangelol E	-7.3
L03	5281672	Myricetin	-8.8
L04	5281806	Psoralidin	-10.2
L05	3008868	Dieckol	-11.4
L06	71659767	Tomentin E	-9.2
L07	3220	Emodin	-8.3
L08	160254	Cryptotanshinone	-8.3
L09	5316743	Dihydrotanshinone l,	-8.1
L10	689043	Caffeic acid	-7.1
L11	1794427	Chlorogenic acid	-9.4
L12	370	Gallic acid	-6.2
L13	5281697	Scutellarein	-8.00
L14	5281600	Amentoflavone	-10.8
L15	102335850	Terrestrimine	-7.3
L16	5280544	Herbacetin	-7.7
L17	168849	Pectolinarin	-10.1
L18	5282150	Rhoifolin	-10.0
L19	145996610	Molnupiravir (Standard)	-7.2

The docking methodology was mainly performed to identify the binding energy of drugs with targeted pathogens (Kumer et al., 2021a; Kumer et al., 2021b; Rahman et al., 2022). So, the PyRx application in the function of AutoDock vina was utilized to perform the molecular docking as it was the most authentic application to calculate the binding energy (Kumer et al., 2021b; Pawar and Rohane, 2021; Kumer et al., 2022).

Normally, -6.00 kcal/mol has been regarded as the potential binding energy for active drugs (Kumer et al., 2022; Sarkar et al., 2022). In the main body of this manuscript, the reported binding energy has been shownt to be better than -6.00 kcal/mol and clearly explained.

The second question which you have mentioned is about the software use in ADMET prediction. The ADMET prediction is calculated by online free access webtool http://lmmd.ecust.edu.cn/admetsar2 known as ADMETSAR (Cheng et al., 2012; Moon et al., 2017), and it is considered to be the most authentic server for the prediction of ADMET data for any bioactive molecules (Cheng et al., 2020).

To carry out the docking investigation, firstly, a targeted protein wasselected and collected from the protein databank https://www.rcsb.org/structure/7MBG (Berman et al., 2007). Then, the protein was purified and excess water and heteroatom was removed by Pymol software 2021 (Molodenskiy et al., 2021). Secondly, all the ligands L01-L19 were downloaded from PubChem database https://pubchem.ncbi.nlm.nih.gov/ (Xie, 2010) and geometry optimization was performed by the application to obtain a stable configuration with optimum energy (Rayan and Rayan, 2017). Finally, they were saved as pdb format for further investigation such as molecular docking and ADMET.

Secondly, the reported Ligand (L05) Dieckol (Yoon et al., 2020), (L14) Amentoflavone (Kim et al., 1998), (L17) Pectolinarin (Cheriet et al., 2020), (L18) Rhoifolin (Xiong et al., 2021) all belong to classes of flavonoid. As they are the same classes of compounds, this could be probable reason for their similar or nearly similar energy binding.

### 14.2 ADMET properties and aquatic and non-aquatic toxicity

The ADME investigations of substances L01-L19 were completed with the help of in-silico techniques (AdmetSAR), and predicted the substances’ absorptions, distributions, metabolisms, and excretions. The findings of the ADME studies are displayed in **Table 4**. The molecules (L01-L19) have a positive Human intestinal absorption, indicating that they can be absorbed gastrointestinally. All of the synthesized molecules were found at a subcellular location in Mitochondria.

**Table 4:** ADMET properties and aquatic and non-aquatic toxicity data.

S/N	PubChem ID	Molecular weight	Human intestinal absorption	Human oral bioavailability	Water solubility logS	Subcellular localization	AMES Toxicity	Carcinogenicity	Acute oral toxicity (kg/mol)
L01	637394	328.36	High	0.55	-2.945	Mitochondria	No	No	0.7662
L02	10022050	370.40	High	0.55	-3.9182	Mitochondria	No	No	0.6048
L03	5281672	318.24	High	0.55	-2.9994	Mitochondria	No	No	0.7348
L04	5281806	336.34	High	0.55	-3.8829	Mitochondria	Yes	No	0.4195
L05	3008868	742.55	High	0.55	-3.4532	Mitochondria	No	No	0.5444
L06	71659767	472.50	High	0.55	-4.4823	Mitochondria	No	No	0.3617
L07	3220	270.24	High	0.55	-3.0170	Mitochondria	No	No	0.6654
L08	160254	296.4	High	0.55	-4.1363	Mitochondria	No	No	0.6176
L09	5316743	278.3	High	0.55	-3,3344	Mitochondria	No	No	0.4254
L10	689043	180.16	High	0.55	-1.6939	Mitochondria	No	No	0.8012
L11	1794427	354.3	High	0.55	-2.4572	Mitochondria	No	No	0.6128
L12	370	170.12	High	0.55	-1.0973	Mitochondria	No	No	0.7405
L13	5281697	286.24	High	0.55	-2.9994	Mitochondria	No	No	0.7348
L14	5281600	538.5	High	0.55	-3.3648	Mitochondria	No	No	0.6395
L15	102335850	343.3	High	0.55	-2.9796	Mitochondria	No	No	0.6355
L16	5280544	302.23	High	0.55	-2.9994	Mitochondria	No	No	0.7348
L17	168849	622.6	High	0.55	-2.9062	Mitochondria	No	No	0.6850
L18	5282150	578.5	High	0.55	-2.4406	Mitochondria	No	No	0.6229
L19	145996610	329.31	High	0.55	-2.7387	Mitochondria	No	No	0.6288

The reported molecules have excellent water solubility elimination, indicating that the chemical has a greater affinity for the aqueous phase. Compounds L06 and L08 have the best affinity for water (-4.4823 and -4.1363) while the standard - Molnupiravir has (-2.7387).

Secondy, The active pharmaceutical ingredients (APIs) have a promising possibility for interfering with the environment. They (APIs) may be mixed through patient excretions into the aquatic and non-aquatic environments and during the manufacturing processes and testing in research laboratories (Fent et al., 2006). It is much more important to determine these compounds’ aquatic and non-aquatic effect on the enviroment to protect our ecosystem from adverse effects. It has been reported that all the natural compounds are free from carcinogens which means they have no chance of producing cancer and they are also free from AMES toxicity, excluding L04. Besides, all the medications have a high GI Absorption rate with oral biaavilability 0.55 for all compounds. Subcellular localization for all compounds are Mitochondria while the acute oral toxicity ranges are 0.3617 kg/mol - 0.7662 kg/mol. The ADME table has been clearly displayed in **Table 4**.

### 14.3 Protein-ligand interaction

**Figure 6:**
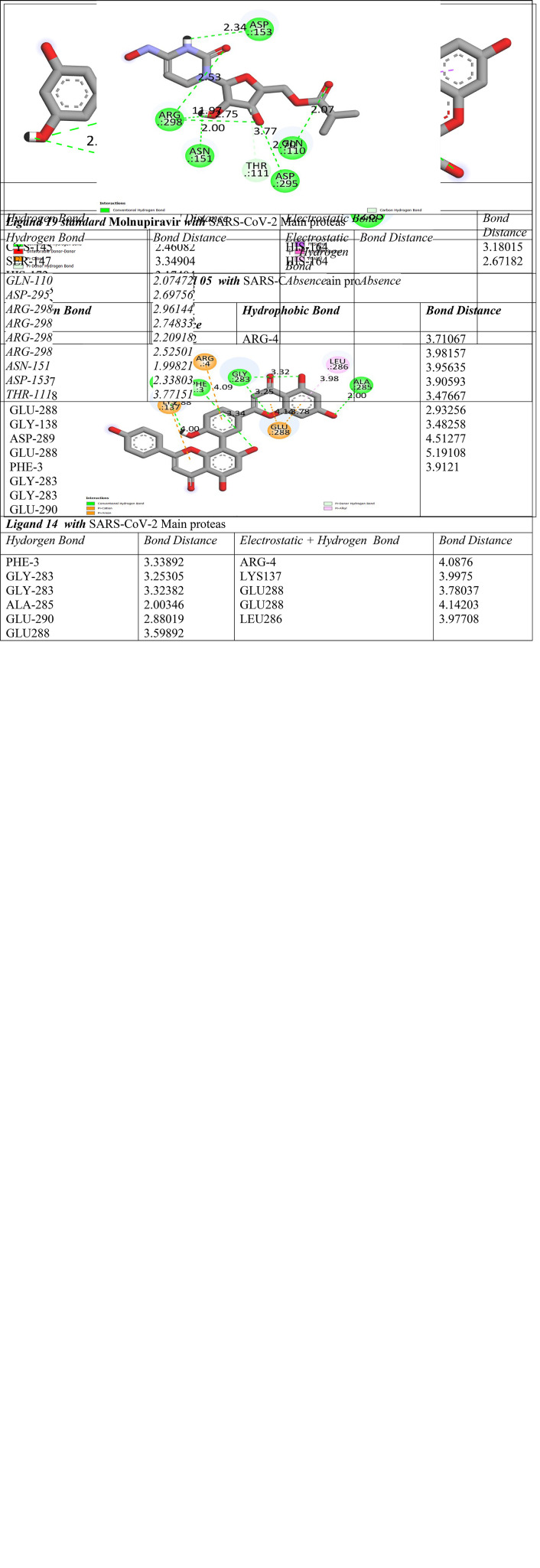
Protein-ligand interaction and their active sides based on High binding energy.

With the help of Discovery Studio, We have determined how many bioactive peptides are present in a protein and how they are connected to a drug or agonist (**Figure 6**). In first step, we performed auto docking on the Protein to determine the binding interactions and inhibit the active site. Our findings have reported three types of bonds present (Hydrogen, Hydrophobic, and electrostatic).

## 15 Conclusion and future perspective

COVID-19 is a severe health disease. Bioactive compounds have been used numerous times to check infections against viral disease and boost the immunological feedback of the host. Valuable natural goods are shown in prior COVID-19 illnesses, including SARS and MERS; therefore, natural ingredients may be suitable and hopeful in this recent outbreak. The immune system of Allium sativum, Camellia sinensis, Zingiber officinale, Nigella sativa, Echinacea spp., and Glycyrrhiza glabra can also increase the immune system and prevent COVID-19. Depending on prior herbal effective therapies for SARS and MERS, sustainable natural medicaments for SARS-CoV-2 are advocated through this fundamental research. Natural substances can reduce COVID-19 at different stages. Several natural substances, such as emodin, reduced S protein, and ACE2 have protected against virus adhesion in a prescribed manner. Several natural chemicals stop copying DNA enzymes from working. The alkaloids homo harringtonine, lycorine, and emetine have an anti-SARS solid CoV-2 effect. SARS-CoV 3CLpro was similarly modestly inhibited by isatin analog. Glycosylated compounds, notably glycosylated terpenoids and terpenoid alkaloids seem to be feasible ways to treat COVID-19, based on the effectiveness of numerous natural medication families. Discovering natural medicines with antiviral action against different types of CoVs like SARS-CoV and MERS-CoV has become an important research target for identifying trearment for the novel COVID-19 virus. The problem could be valuable in increasing and encouraging research projects on helpful natural substances for COVID-19 control and remedy: cleansed natural constituents are researched for their pharmacokinetic and pharmacodynamics characteristics (absorption, distribution, metabolism, and excretion) and also their anti-SARS-CoV-2 properties; suitable quality assurance evaluations for plant extracting constituents which are used as immunological stimulants; discover numerous targets for coronavirus elimination; (chronic and acute toxicological studies). After overall review, we wanted to find the most potent drug among the reported natural compound. So, the computational investigations have been performed against SARS-CoV-2 main Protease. So, molecular docking, properties and aquatic and non-aquatic Toxicity have been studied using an in-silico method. Overall studies show that the most Potent Natural Bioactive compound is Dieckol (docking score -11.4 kcal/mol). The Pharmacokinetics, aquatic, nonaquatic study has reported that all the ligands are free from carcinogenicity excluding L04, and have Acute Oral Toxicity and Good water solubility. COVID-19 has been labeled a public health emergency of worldwide concern by the WHO. SARS-CoV-2 is the eighth member of the family of CoVs that infect humans, belonging to the Orthocoronavirinae subfamily of the Coronaviridae family and Nidovirales order. As a result, gaining a better knowledge of SARS-CoV-2 is critical for developing effective vaccines and treatments (Ding et al., 2019). This in silico investigation was carried out in order to find an effective medication against SARS-CoV-2. For the prediction of novel drug-target relationships, in silico methods such as docking are well-established and experimentally verified. Docking is also well-suited for drug repurposing, whether drug-based or target-based (Martínez‐Campos et al., 2019). The different discoveries achieved by computational methods are significant in terms of deducing a prospective medicine, as evidenced by the success of the derived compounds, and evidenced by the drugs now in use and future ones on the horizon (Blundell et al., 2006). The proposed study suggests that repurposing and using naturally existing compounds could be useful therapies for the treatment of COVID-19. Six molecules were chosen from the entire pool to be explored using different computational algorithms in this computational investigation for the prediction of possible drug-like naturally occurring chemicals against SARS-CoV-2. The six compounds discovered were found to have the ability to bind to the SARS-CoV-2 RdRp protein. As a result, these adaptable natural compounds have the potential to have an inhibitory effect and serve as a novel COVID-19 inhibitor and treatment (Bharti and Shukla., 2021).

As a result of our discoveries using a structural bioinformatics approach, we believe that all of these drugs have the potential to be employed against SARS-CoV-2 and should be investigated further as COVID-19 preventative therapies.

## Authors contributions

MMR, MI, SA, TE, E-SS, and PW conceptualized and designed the manuscript, participating in drafting the article and/or acquisition of data, and/or analysis and interpretation of data; EA, SM, MSR, RS, FA, SHS, MH, and TR prepared the figures and tables. TE, EA, SS, MA, E-SS, and PW wrote, edited and revised the manuscript critically. TE and PW revised the final written. All authors critically revised the manuscript concerning intellectual content and approved the final manuscript.

## Funding

This publication was supported by the Deanship of Scientific Research at Prince Sattam Bin Abdelaziz University, Al- Kharj, Saudi Arabia as well as the authors are sincerely grateful to Egyptian Russian University, Badr, Egypt.

## Acknowledgments

E-SS thanks, Proyecto Interno I+D UCEN (CIP2020036) for the financial support to this contribution.

## Conflict of interest

The authors declare that the research was conducted in the absence of any commercial or financial relationships that could be construed as a potential conflict of interest.

## Publisher’s note

All claims expressed in this article are solely those of the authors and do not necessarily represent those of their affiliated organizations, or those of the publisher, the editors and the reviewers. Any product that may be evaluated in this article, or claim that may be made by its manufacturer, is not guaranteed or endorsed by the publisher.

## Abbreviations

RNA, Ribo nucleic acid; RdRp, rna dependent rna polymerase; AMES; ACE2, angiotensine converting enzyme; TMPRSS, receptors and transmembrane protease serine 2; CADD, computer aided drug discovery; MERS, middle east respiratory syndrome coronavirus; Nsps, encodes sixteen nonstructural proteins; RAAS, Renin Angiotensin Aldosterone System; AT1R, angiotensin receptor type 1; RTC; CPE, cytopathic impact examine; FFM1 & FFM2; SAR, structure activity relationship; MAPK, mitogen-activated protein kinase; NF-kB; Nrf2; PTGS2, prostaglandin-endoperoxide synthase 2; Bcl-2, B-cell lymphoma 2; ORF3a, open reading frame 3a; PIK3CG, phosphatidylinositol-4,5-bisphosphate 3-kinase catalytic subunit gamma; JAK/STAT; TCM, Traditional Chinese medicines; QPD, Qingfey Pay Decoction; CPM, Chainese patent medicine; TCMS; AdmetSAR.
